# Nature-Derived Ferulic Acid Hybrids with Enhanced Antifungal and Antivirulence Activity Against *Candida albicans*

**DOI:** 10.3390/ijms27062859

**Published:** 2026-03-21

**Authors:** Dylan Lambert, Celia Lemaire, Louis Camaioni, Muriel Billamboz, Samir Jawhara

**Affiliations:** 1 CNRS, UMR 8576-UGSF—Unité de Glycobiologie Structurale et Fonctionnelle, INSERM U1285, F-59000 Lille, France; dylan.lambert2.etu@univ-lille.fr (D.L.); celia.lemaire@univ-lille.fr (C.L.); louis.camaioni@gmail.com (L.C.); 2 Medicine Faculty, University of Lille, F-59000 Lille, France; 3 CHU Lille, Service de Parasitologie Mycologie, Pôle de Biologie Pathologie Génétique, F-59000 Lille, France; 4 ICL, JUNIA, Université Catholique de Lille, LITL, F-59000 Lille, France; muriel.billamboz@junia.com

**Keywords:** *Candida albicans*, antifungal agents, natural products, ferulic acid derivatives, bio-based hybrid molecules, anti-virulence activity, structure–activity relationship, biofilm inhibition, mitochondrial dysfunction

## Abstract

The high incidence of *Candida albicans* infections and the limited efficacy of current antifungal therapies highlight the need for new antifungal agents. In this study, we present a bio-based hybridization strategy aimed at enhancing the antifungal activity of natural product scaffolds, with a particular focus on *trans*-ferulic acid. A library of twenty-nine hybrid molecules was rationally generated by grafting naturally occurring lipophilic moieties onto either the phenolic or carboxylic acid functions of ferulic acid. The antifungal activity of these molecules was then assessed against *C. albicans*. While the parent natural compounds exhibited weak activity (MIC > 500 µM), several hybrid derivatives (ATF19, ATF20, and MB22) demonstrated enhanced potency, with MIC values of <50 µM. Esters of the carboxylic acid or phenol group were essential for activity, with the most potent effects observed for short linear or mildly branched lipophilic chains. These active compounds exerted a multifaceted anti-virulence effect, including mitochondrial membrane depolarization, inhibition of hyphal morphogenesis, alterations in cell wall composition, and strong suppression of biofilm formation. Additionally, lead compounds showed no detectable cytotoxicity in human macrophages and intestinal epithelial cells and significantly improved host survival in a *Caenorhabditis elegans* model of *C. albicans* infection. Overall, the ferulic acid, citronellol, and sinapic hybrid molecules emerged as promising lead compounds for the development of antifungals against *C. albicans*.

## 1. Introduction

The increasing prevalence of drug-resistant *Candida albicans* strains highlights the urgent need to expand and strengthen the current therapeutic arsenal with new antifungal agents [1,2,3]. In this context, natural or bio-based compounds are particularly attractive candidates due to their unique combination of biological efficacy and environmental compatibility [4,5]. Unlike purely synthetic molecules, bio-based compounds are generally derived from renewable resources, rapidly biodegrade, and pose a minimal ecological burden. Their sustainable production and reduced persistence in the environment make them particularly advantageous for long-term use, as they mitigate the risks of chemical accumulation and environmental toxicity. Furthermore, their mechanisms of action often reduce the likelihood of resistance development in pathogens, enhancing their appeal as antifungal agents. Phenolic compounds, which are widely studied as secondary metabolites naturally abundant in numerous plant species, are among this diverse class [6]. Beyond their longstanding industrial applications in flavors, spices, cosmetics, and food additives, phenolic compounds are increasingly recognized for their broad antimicrobial properties [6].

*Trans-ferulic* acid emerges as a highly promising bioactive molecule within this chemical space. It consists of a phenolic aromatic ring that is substituted with a methoxy group in position 3 and is linked to an acrylic acid moiety in position 1. This compound is ubiquitous in the plant kingdom, particularly in the seeds and leaves of fruits, vegetables, and cereals, where it contributes to cell wall integrity by forming covalent bonds between polysaccharides and lignin [7,8]. Its wide availability, low cost, and natural origin make it an attractive and environmentally sustainable resource for further drug development.

*Trans*-ferulic acid exhibits broad-spectrum antimicrobial activity against both Gram-positive bacteria, such as *Bacillus subtilis*, *Listeria monocytogenes*, and *Staphylococcus aureus*, and Gram-negative bacteria, including *Escherichia coli*, *Pseudomonas aeruginosa*, and *Salmonella paratyphi*, with minimum inhibitory concentrations ranging from tens to hundreds of milligrams per liter [9]. Its moderate hydrophobicity (log*P* = 1.36) enables it to penetrate microbial membranes, inducing changes in membrane hydrophilicity, surface charge, and cytoplasmic permeability and, ultimately, disrupting cellular homeostasis [9].

Based on this rationale, the present study aimed to design and evaluate hybrid bio-based molecules derived from *trans*-ferulic acid as potential antifungal candidates against *C. albicans*. The effects of these molecules on Candida viability, cell wall integrity, filamentation, biofilm formation, and activity against clinical isolates resistant to conventional antifungal drugs were assessed. Additionally, their efficacy was tested using an in vivo *Caenorhabditis elegans* infection model to provide an integrated evaluation of antifungal potential and host–pathogen interactions. By developing ferulic acid-derived hybrids, this study seeks to advance the creation of sustainable, environmentally friendly, and highly effective antifungal agents that address the urgent challenge of multidrug-resistant *C. albicans* infections while minimizing ecological impact.

## 2. Results

Hybrid bio-based molecules derived from *trans*-ferulic acid were obtained as potential antifungal agents against *C. albicans* by chemically modifying the acid to introduce lipophilic moieties on either the phenolic group (sub-family F1–F8 and MB22) or on the carboxylic acid function (sub-family F9-ATF20). The lipophilic fragments were selected from naturally occurring compounds and consisted mainly of short- to medium-chain fatty acids. Additional natural molecules, including citronellol, (-)-menthol, and kojic acid, were also incorporated. Similarly, sinapic acid and isoeugenol were derivatized to evaluate the performance of ferulic acid-based analogs. The antifungal activity of all the compounds was evaluated by determining their minimum inhibitory concentrations (MICs) against a susceptible strain of *C. albicans*. All synthesized molecules and their respective MIC values are summarized in Table 1. In total, twenty-nine compounds were investigated. As shown in Table 1, none of the natural platform molecules (ferulic acid, citronellol, kojic acid, (-)-menthol, sinapic acid, and isoeugenol) exhibited significant antifungal activity, with MIC values of 500 µM or greater. By contrast, coupling these natural scaffolds either together or with fatty acids resulted in the identification of several compounds with markedly improved activity, with MIC values of ≤50 µM. For comparison, the reference antifungal drug fluconazole exhibited an MIC of 0.5 µg/mL under the same conditions. As fluconazole is a fully synthetic molecule, it was used only as a reference control and was not included among the bio-based hybrid molecules evaluated in Table 1.

The derivatization of the phenolic moiety with C2–C9 fatty acids (see Table 1, entries 2–9) provided valuable information. Most of these compounds exhibited improved activity compared with ferulic acid itself, with the exception of the butanoic acid derivative F4. These results suggest that the free phenolic group is not essential for antifungal activity. Furthermore, overall lipophilicity does not appear to be a determining factor, as no clear correlation was observed between activity and the length of the linear alkyl chain. From this series, MB22, bearing a propylate moiety, showed the highest potential with MIC = 10 µM against *C. albicans*.

By contrast, esterification of the carboxylic acid function of ferulic acid revealed a clear structure–activity relationship. Optimal antifungal activity was observed for derivatives bearing C6–C8 linear aliphatic chains (Table 1, entries 15–17). This trend was further supported by the incorporation of citronellol, which has a branched unsaturated C8 chain. Compound ATF20, resulting from the coupling of ferulic acid and citronellol, was one of the most active molecules identified. Conversely, the introduction of (-)-menthol, a cyclic analog of citronellol, led to a pronounced decrease in activity (Table 1, entry 20; compound F19), indicating a preference for linear lipophilic substituents. This observation is consistent with the weak activity of the kojic acid–ferulic acid hybrid F18.

Compound ATF19, obtained by esterification of sinapic acid with pentan-1-ol, is the direct analog of compound F13 (Table 1, entry 28). ATF19 exhibited a better MIC value, indicating that the additional methoxy group on the aromatic ring of sinapic acid could help improve the antifungal activity. Similarly, compound E2, derived from isoeugenol, can be considered an analog of F2 lacking the carboxylic acid function (Table 1, entry 29). The MIC values of these two compounds were comparable (47 µM vs. 56 µM), suggesting that the carboxylic acid moiety is not critical for activity.

These results demonstrate that chemical coupling of natural product scaffolds is an effective strategy to enhance antifungal activity against *C. albicans*. Based on their potency and structural features, compounds MB22, ATF19, and ATF20, derived from ferulic acid, sinapic acid, fatty acids, and citronellol, were selected for further investigation.

### 2.1. Cytotoxicity Assessment of Compounds in Macrophage and Intestinal Epithelial Cell Models

**Figure 1:**
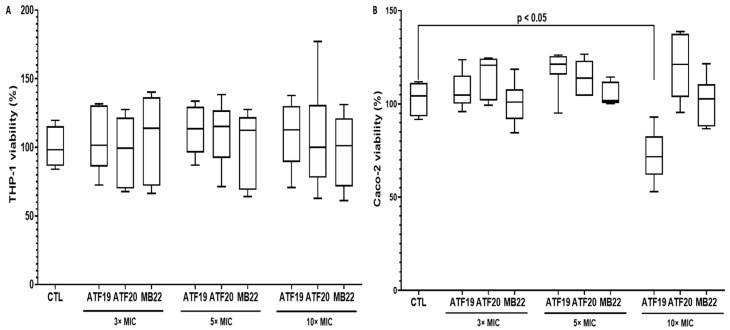
Cytotoxicity evaluation of compounds ATF19, ATF20, and MB22 in human macrophage and intestinal epithelial cell models. (**A**,**B**) Differentiated THP-1 macrophages and human intestinal epithelial Caco-2 cells were exposed for 24 h to compounds ATF19, ATF20, or MB22 at concentrations corresponding to 3×, 5×, or 10× MIC. Cell viability was assessed using an MTT-based assay and expressed relative to untreated control cells (CTL). In THP-1 macrophages (**A**), none of the tested compounds induced a significant reduction in cell viability across the range of concentrations evaluated. In Caco-2 cells (**B**), compounds ATF20 and MB22 did not significantly affect cell viability at any concentration tested, whereas exposure to compound ATF19 at 10× MIC resulted in a moderate but statistically significant decrease in cell survival. Data are presented as mean ± SD of eight independent replicates (*n* = 8).

In order to evaluate the potential cytotoxic effects of the compounds ATF19, ATF20, and MB22 in vitro, differentiated THP-1 macrophages were exposed to increasing concentrations corresponding to 3×, 5×, and 10× the MIC. After 24 h of exposure, none of the tested compounds induced detectable cytotoxicity in THP-1 macrophages. Cell viability remained close to 100% across all concentrations tested, indicating a favorable safety profile in this immune cell model (Figure 1A).

The compounds’ cytotoxic potential was also assessed in human intestinal epithelial Caco-2 cells using the same experimental design. Overall, compounds ATF19, ATF20, and MB22 did not significantly affect Caco-2 cell viability at concentrations up to five times the MIC, with survival rates remaining near baseline levels after 24 h of treatment. However, exposure to compound ATF19 at 10× MIC resulted in a significant reduction in cell viability, with only around 75% of cells remaining viable after 24 h. This suggests that compound ATF19 may exert cytotoxic effects on intestinal epithelial cells at supra-therapeutic concentrations (Figure 1B).

### 2.2. Effects of Compounds on C. albicans Cellular Integrity

**Figure 2:**
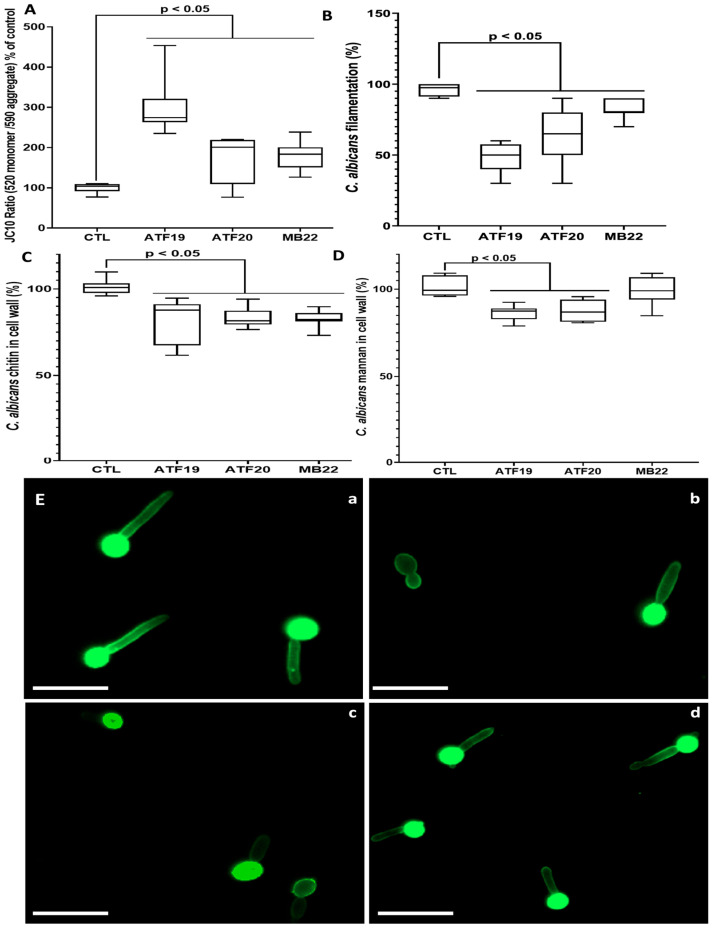
Effects of compounds ATF19, ATF20, and MB22 on *C. albicans* cellular integrity and virulence-associated traits. (**A**) Mitochondrial membrane potential of *C. albicans* following treatment with compounds ATF19, ATF20, or MB22 at 1× MIC for 24 h, assessed by the JC-10 monomer-to-aggregate fluorescence ratio. Untreated yeast cells served as control (CTL). (**B**) Quantification of filamentation in *C. albicans* after 2 h of exposure to the compounds at 1× MIC. Results are expressed as the percentage of filamentous cells relative to the total population. (**C**) Chitin content of the *C. albicans* cell wall following treatment at 1× MIC, expressed relative to untreated controls. (**D**) Mannan content of the fungal cell wall was measured after compound exposure at 1× MIC. (**E**) Representative microscopic images illustrating the effects of the compounds on *C. albicans* morphology and cell wall mannans following Galanthus nivalis lectin (GNL) staining: untreated cells (**a**); cells treated with compound ATF19 (**b**), compound ATF20 (**c**), or compound MB22 (**d**) at 1× MIC. Scale bar: 10 µm. Data represent the mean ± SD with *n* = 6 (**A**), *n* = 8 (**B**), and *n* = 12 (**C**,**D**).

Several parameters were examined to determine whether compounds ATF19, ATF20, and MB22 affect the structural integrity of *C. albicans*. First, mitochondrial membrane potential was assessed using the JC-10 assay following treatment with each compound at 1× MIC. All treatments resulted in a marked and statistically significant increase in the JC-10 monomer-to-aggregate fluorescence ratio after 24 h, indicating mitochondrial depolarization. The observed increase ranged from approximately 200% to 300% relative to untreated controls. Of the tested compounds, ATF19 had the most pronounced effect, with the JC-10 ratio reaching nearly 300% of control values (Figure 2A). In addition to mitochondrial depolarization, we evaluated the production of ROS in *C. albicans* following treatment with the selected compounds. The results showed that treatment with compound ATF19 did not induce a significant increase in ROS production after 6 h of incubation. In contrast, compounds ATF20 and MB22 led to a noticeable increase in ROS levels under the same conditions (in Supporting Information).

Given the importance of filamentation as a major virulence trait of *C. albicans*, the impact of the compounds on hyphal development was next evaluated. Treatment with compounds ATF19, ATF20, or MB22 at 1× MIC significantly reduced filamentation after 2 h of incubation. Quantitative analysis revealed a 20–50% decrease in the proportion of filamentous cells compared to the untreated condition. Consistent with mitochondrial data, ATF19 was found to be the most inhibitory, reducing filamentation by around 50% (Figure 2B).

Further investigations of alterations in fungal cell wall composition involved quantifying chitin and mannan levels following exposure to compounds. Treatment with compounds ATF19, ATF20, and MB22 at 1× MIC resulted in a significant reduction in chitin content of between 10% and 20%, compared to untreated *C. albicans* cells (see Figure 2C). Concurrently, a notable decrease in mannan levels (approximately 15%) was observed following treatment with compounds ATF19 and ATF20. However, MB22 treatment did not result in a statistically significant change in mannan content relative to controls (Figure 2D).

To visually corroborate these quantitative findings, fluorescence and light microscopy analyses were performed after treatment at 1× MIC and staining with GNL. Untreated *C. albicans* cells exhibited extensive hyphal elongation and strong green fluorescence, reflecting abundant mannan deposition within the cell wall (Figure 2E, panel a). In contrast, compound-treated cells displayed shorter filaments than controls (Figure 2E, panels b–d). Furthermore, treatment with compounds ATF19 and ATF20 resulted in reduced GNL fluorescence intensity, consistent with the observed decrease in mannan content (Figure 2E, panels b and c).

### 2.3. Impact of Compounds on C. albicans Biofilm Formation

**Figure 3:**
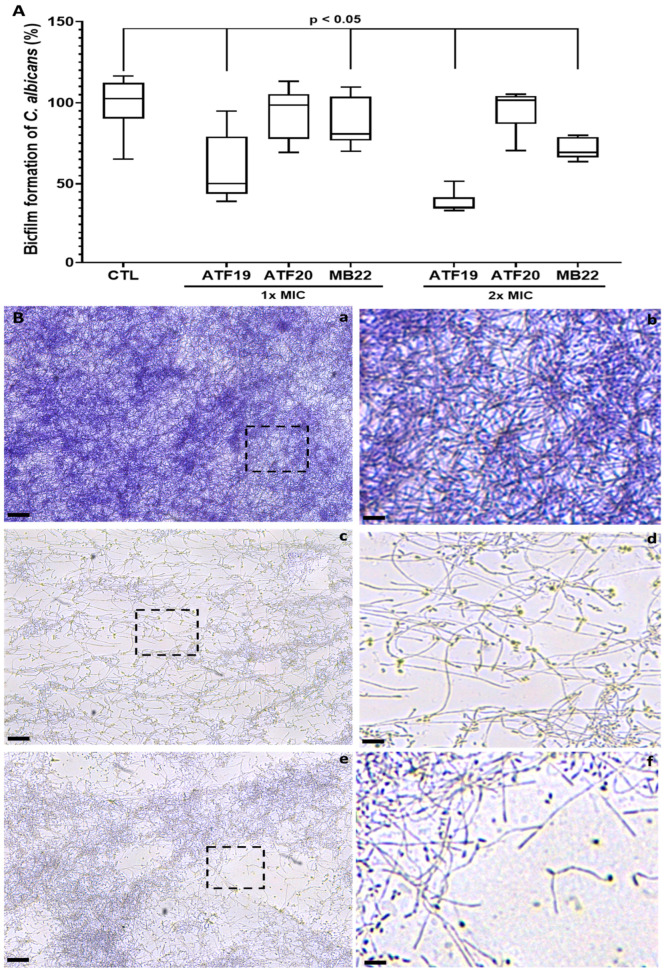
Inhibitory effects of compounds ATF19, ATF20, and MB22 on *C. albicans* biofilm formation. (**A**) Quantification of biofilm biomass following treatment with compounds ATF19, ATF20, or MB22 at 1× MIC, and with compounds ATF19 and MB22 at 2× MIC. Biofilm formation was assessed after 24 h using crystal violet staining and expressed relative to untreated controls (CTL). Data represent the mean ± SD of eight independent experiments (*n* = 8). (**B**) Representative microscopic images of *C. albicans* biofilms formed under control conditions (**a**,**b**) or following treatment with compound ATF19 at 2× MIC (**c**,**d**) or compound MB22 at 2× MIC (**e**,**f**). The dashed boxes correspond to large-scale images. Scale bars correspond to 200 µm (**a**,**c**,**e**) and 50 µm (**b**,**d**,**f**).

The effects of the compounds on biofilm formation, which is another key virulence factor in *C. albicans*, were then examined. Yeast cells were treated with compounds ATF19, ATF20, and MB22 at 1× MIC, as well as with compounds ATF19 and MB22 at 2× MIC. Treatment with compounds ATF19 and MB22 at 1× MIC significantly impaired biofilm formation after 24 h, resulting in reductions of approximately 50% and 15%, respectively, compared to untreated controls. Increasing the concentration to 2× MIC further enhanced this inhibitory effect, reducing biofilm biomass by approximately 65% and 30% for compounds ATF19 and MB22, respectively. Overall, compound ATF19 consistently exhibited the strongest antibiofilm activity. Treatment with compound ATF20 at 1× MIC showed a tendency towards reducing biofilms, although this effect was not statistically significant (Figure 3A). Microscopic visualization following crystal violet staining corroborated these quantitative results. Untreated *C. albicans* formed dense and homogeneous biofilms that covered the entire surface of the well (Figure 3B, panels a and b). In contrast, treatment with compounds ATF19 or MB22 at 2× MIC led to a substantial disruption of biofilm architecture, characterized by reduced staining intensity and the presence of large voids within the biofilm matrix. These images clearly demonstrate the more pronounced antibiofilm effect of compound ATF19 relative to MB22 (Figure 3B, panels c–f).

### 2.4. Effects of Compounds on C. elegans Survival Following C. albicans Infection

**Figure 4:**
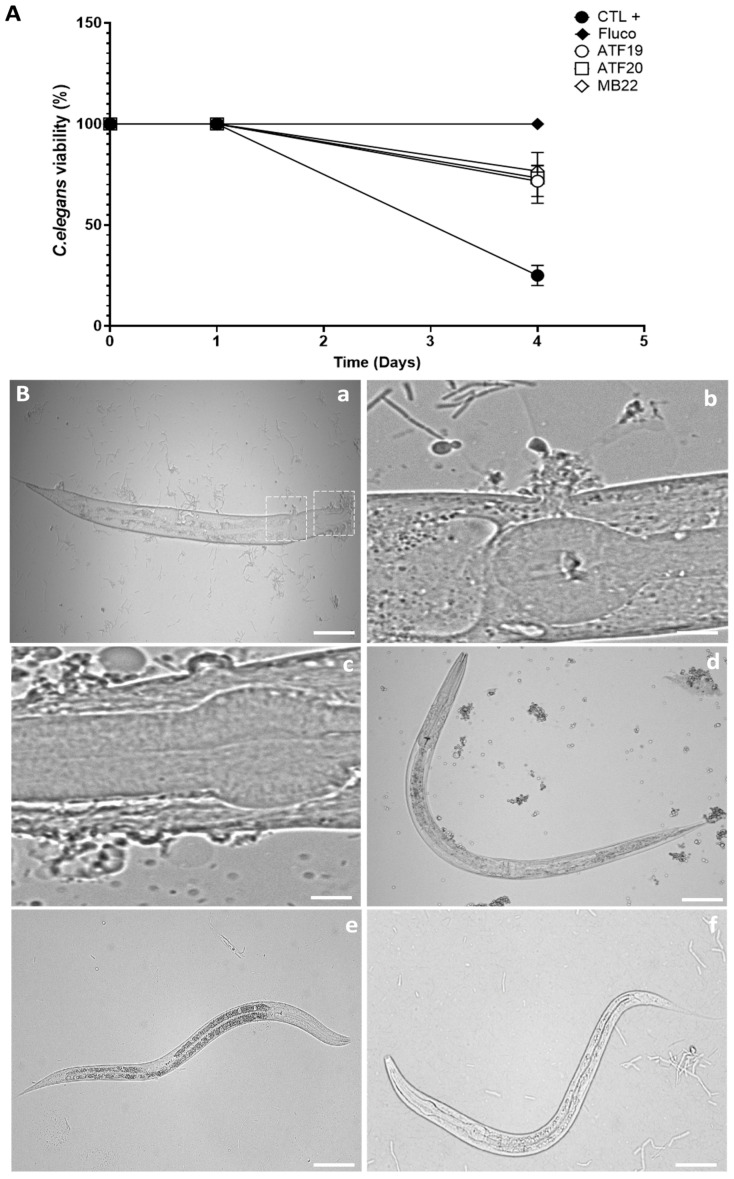
Protective effects of compounds ATF19, ATF20, and MB22 in a *C. elegans* model of *C. albicans* infection. (**A**) Survival analysis of *C. elegans* infected with *C. albicans* and treated with compounds ATF19, ATF20, or MB22 at 1× MIC. Infected but untreated nematodes served as the positive control (CTL+). Survival was monitored daily, and results are expressed as the percentage of surviving worms. Data represent the mean ± SD from nine independent experiments (*n* = 9). (**B**) Representative microscopic images of *C. elegans* following *C. albicans* infection. Untreated infected nematodes show extensive tissue damage and lethality (**a**–**c**), whereas infected nematodes treated with compound ATF19 (**d**), compound ATF20 (**e**), or compound MB22 (**f**) exhibit preserved morphology and survival. The two dashed boxes in image (**a**) correspond to the large-scale images shown in (**b**) and (**c**). Scale bars: 200 µm (**a**,**d**,**e**,**f**) and 20 µm (**b**,**c**).

To evaluate the protective effects of the compounds in the context of a host–pathogen interaction, their efficacy was assessed using a *C. elegans* model of *C. albicans* infection. Treatment of nematodes with fluconazole at 1× MIC protected the nematodes, resulting in 100% survival at day 4. Infected nematodes were treated with the compounds ATF19, ATF20, or MB22 at a concentration of 1× MIC, and their survival was monitored over time. Treatment with any of the three compounds resulted in a substantial improvement in host survival. Around 75% of the treated nematodes remained alive four days after infection, compared to a survival rate of around 25% in the infected, untreated control group (see Figure 4A). Microscopic examination at the end of the experiment provided further evidence of the protective effects of the compounds. Untreated infected nematodes did not survive and exhibited multiple cuticular perforations, which are consistent with the invasive hyphal penetration and dissemination of *C. albicans* hyphae (Figure 4B, panels a–c). By contrast, nematodes infected with *C. albicans* and treated with ATF19, ATF20, or MB22 survived the infection, showing no visible signs of tissue perforation or structural damage (Figure 4B, panels d–f).

## 3. Discussion

The increasing incidence of *C. albicans* infections, together with the emergence of resistance to existing antifungal agents and the frequent toxicity issues associated with them, highlights the urgent need for novel, safe, and effective antifungal strategies. Against this backdrop, the present study explores a rational, bio-inspired approach based on the chemical coupling of natural product scaffolds to generate hybrid molecules with enhanced antifungal activity and reduced host toxicity. Our results demonstrate that the strategic derivatization of *trans*-ferulic acid with naturally occurring lipophilic moieties yields compounds with markedly improved antifungal potency, significant anti-virulence properties, and favorable safety profiles.

A key finding of this study is that individual natural platform molecules (ferulic acid, citronellol, kojic acid, (-)-menthol, sinapic acid, and isoeugenol) exhibited little to no antifungal activity when tested alone. This finding is consistent with previous reports describing the modest antimicrobial effects of phenolic acids and terpenoids, which are often limited by poor membrane penetration or insufficient intracellular targeting [10,11]. It is important to note that natural compounds such as ferulic acid generally display moderate antifungal activity compared to standard agents, such as fluconazole or amphotericin B, and are primarily investigated for their complementary or anti-virulence properties [9].

However, when these scaffolds were chemically coupled either to each other or to fatty acid chains, a clear increase in antifungal potency was observed, with several derivatives displaying MIC values ≤ 50 µM. These results strongly support the concept that hybridization of natural products can unlock synergistic or emergent bioactivities that cannot be achieved with the parent compounds alone [12,13].

Systematic structure–activity relationship analysis revealed that the site of derivatization on ferulic acid plays a critical role in determining its antifungal efficacy [12,13]. Modification of the phenolic hydroxyl group with short- to medium-chain fatty acids generally resulted in better activity, with MB22, the propyl derivative, being the most potent antifungal. Moreover, the lack of correlation between alkyl chain length and activity in this sub-series suggests that simple increases in lipophilicity are insufficient to drive potency, underscoring the importance of more subtle structural features [14].

In contrast, esterification of the carboxylic acid function of ferulic acid yielded a pronounced and well-defined activity profile. Derivatives bearing C6–C8 linear aliphatic chains exhibited optimal antifungal activity, indicating the existence of a favorable balance between hydrophobicity, molecular flexibility, and biological accessibility [15,16]. This optimal chain length likely facilitates efficient interaction with fungal membranes while avoiding excessive hydrophobicity that could impair solubility or selectivity. The high activity of the citronellol-ferulic acid hybrid (ATF20), which incorporates a branched unsaturated C8 chain, further supports this hypothesis and suggests that limited branching and unsaturation can be accommodated without loss of efficacy.

Conversely, the sharp decrease in activity observed upon the incorporation of (-)-menthol, a bulky cyclic analog of citronellol, highlights the importance of molecular shape and conformational flexibility [17,18]. Similarly, the poor performance of the kojic acid–ferulic acid hybrid suggests that excessive polarity or rigid aromatic frameworks are detrimental to antifungal activity [19,20]. Together, these observations suggest that linear or mildly branched lipophilic moieties are preferred, probably because they facilitate productive interactions with fungal lipid bilayers or membrane-associated targets.

Comparative analyses using sinapic acid and isoeugenol derivatives provided additional mechanistic insight. The different MIC values observed for sinapic acid and ferulic acid analogs differing only by an extra methoxy group indicate that increased aromatic substitution could help enhance antifungal potency [21,22]. ATF19 displayed the same activity as F6, which displayed the same overall lipophilicity, suggesting the importance of this balance. Similarly, the comparable activity of isoeugenol-derived compounds lacking a carboxylic acid function suggests that this moiety is not critical for antifungal action. Taken together, these findings argue against a single, highly specific enzymatic target, instead supporting a mechanism involving membrane perturbation and downstream cellular dysfunction.

Consistent with this hypothesis, mechanistic investigations revealed that the most active compounds induce significant changes to key cellular processes in *C. albicans*. All of the tested lead compounds triggered significant mitochondrial membrane depolarization, indicating the early disruption of fungal energy homeostasis [23,24]. Mitochondrial dysfunction is increasingly recognized as a potent antifungal mechanism, as it can simultaneously impair metabolism, stress responses, and morphogenetic programs. Consistent with this, the compounds also significantly inhibited the yeast-to-hypha transition, a central virulence trait of *C. albicans* that is required for tissue invasion and immune evasion [25,26]. In addition, ROS production assays showed that ATF19 did not significantly increase ROS levels after 6 h, whereas ATF20 and MB22 induced a marked increase under the same conditions. These results suggest that mitochondrial depolarization induced by ATF19 may occur through a mechanism independent of early ROS overproduction, whereas ATF20 and MB22 appear to induce oxidative stress in *C. albicans*. This indicates that the relationship between mitochondrial dysfunction and ROS generation may vary depending on the compound and its mode of action, as previously reported in fungal systems, where ROS production can differ in timing and magnitude depending on the stressor [27].

Furthermore, treatment with the lead compounds resulted in measurable reductions in cell wall chitin and mannan content, key structural and immunologically relevant components of the fungal cell wall [28]. Alterations in cell wall architecture not only compromise fungal integrity and sensitize cells to environmental stress and host immune defenses [29,30]. Microscopic analyses corroborated these biochemical findings, revealing shortened hyphae and reduced mannan deposition following exposure to the compounds. Importantly, these effects were observed at concentrations corresponding to the MIC, indicating that anti-virulence activity is closely linked to antifungal efficacy rather than being a secondary consequence of growth inhibition.

The compounds’ ability to interfere with biofilm formation further reinforces their therapeutic potential. Biofilms present a major clinical challenge due to their intrinsic resistance to antifungal drugs and the host immune response. Compound ATF19 exhibits particularly strong antibiofilm activity, suggesting that these hybrid molecules may be effective against both planktonic cells and structured fungal communities [31,32,33]. This property is particularly important for treating device-associated and mucosal candidiasis.

Equally important, the lead compounds exhibited a favorable safety profile in vitro. No cytotoxicity was detected in macrophages or intestinal epithelial cells at concentrations up to five times the MIC, and only limited toxicity was observed at supra-therapeutic concentrations for one compound [34,35]. These results indicate a promising therapeutic window and suggest that selective targeting of fungal cells can be achieved without compromising host cell viability.

Finally, the protective effects observed in the *C. elegans* infection model provide compelling in vivo support for the antifungal and anti-virulence activities of these compounds [20,36,37]. Treatment significantly improved host survival and prevented hyphal-mediated tissue damage, demonstrating that the compounds retain efficacy in a complex host–pathogen context. The concordance between in vitro mechanistic data and in vivo outcomes strongly supports the translational relevance of this approach.

## 4. Materials and Methods

### 4.1. C. albicans Strain and Culture Conditions

*C. albicans* reference strain SC5314 (wild type; ATCC MYA-2876) was used throughout this study [38]. Yeast cells were routinely maintained on Sabouraud Dextrose Agar (SDA) plates and incubated at 37 °C for 24 h. For experimental assays, colonies were harvested and resuspended in liquid Sabouraud medium, followed by incubation at 37 °C under shaking conditions. Actively growing *C. albicans* cultures were subsequently used for antifungal susceptibility and phenotypic analyses.

### 4.2. Chemical Compounds and Cell Culture Models

The synthetic compounds designated ATF19, ATF20, and MB22 were synthesized and provided by JUNIA-HEI (Lille, France). All procedures and descriptions were added as Supporting Information. Compounds were chemically characterized prior to biological evaluation. These compounds were obtained through single-step syntheses followed by purification, in contrast to the multi-step procedures typically required for commercially available antifungal agents. The stability of selected compounds was evaluated in pure water (pH 6.5) at a concentration of 1 g/L. After one week at room temperature, no degradation of these compounds was observed under these conditions. Their antifungal activities were assessed using both in vitro and in vivo experimental models involving *C. albicans*.

The human monocytic THP-1 cell line (ATCC TIB-202) was cultured in RPMI-1640 medium (Fisher Scientific, Illkirch, France) supplemented with 10% fetal bovine serum (FBS; Sigma-Aldrich, St. Quentin Fallavier, France) and 1% penicillin–streptomycin (Fisher Scientific) [20,39]. Human intestinal epithelial Caco-2 cells were maintained in DMEM (Fisher Scientific) supplemented with 20% FBS and 1% penicillin–streptomycin. All cell cultures were incubated at 37 °C in a humidified atmosphere containing 5% CO_2_.

### 4.3. Antifungal Activity and Determination of Minimum Inhibitory Concentration (MIC)

The effects of compounds ATF19, ATF20, and MB22 on *C. albicans* viability and growth were evaluated using decreasing concentrations. Minimum inhibitory concentrations (MICs) were determined using an Alamar Blue–based assay. Briefly, 5 × 10^3^ yeast cells were dispensed into each well of a transparent 96-well plate (Greiner Bio-One, 655101, Paris, France) containing 90 µL of RPMI medium. Alamar Blue reagent (10 µL; Thermo Fisher Scientific, Illkirch-Graffenstaden, France) was added, followed by the compounds at concentrations ranging from 5 × 10^−3^ M to 5 × 10^−6^ M [20].

MIC values were defined as the lowest compound concentration resulting in ≥95% inhibition of fungal growth. Optical density measurements were recorded at 600 nm using a FLUOstar microplate reader (BMG Labtech, Champigny-sur-Marne, France) at baseline (T_0_) and after 24 h of incubation (T_24_).

### 4.4. Cytotoxicity Assessment in Macrophage and Intestinal Epithelial Cell Models

THP-1 monocytes were differentiated into macrophages by treatment with phorbol-12-myristate-13-acetate (PMA; 200 ng/mL; Sigma-Aldrich, St. Quentin Fallavier, France) for 72 h at 37 °C in 5% CO_2_. Differentiated THP-1 macrophages and Caco-2 cells were seeded at a density of 1 × 10^5^ cells/mL in transparent 96-well plates and exposed to compounds ATF19, ATF20, or MB22 at concentrations corresponding to 3×, 5×, and 10× the MIC. Cells were incubated for 24 h at 37 °C in 5% CO_2_ [37].

Cell viability was evaluated using the MTT assay [20,40]. Following incubation, 10 µL of MTT reagent (3-(4,5-dimethylthiazol-2-yl)-2,5-diphenyltetrazolium bromide; Bio-Techne, France) was added to each well, and the plates were incubated for an additional 4 h. Formazan crystals were solubilized using 100 µL of MTT detergent solution, and absorbance was measured at 570 nm to assess cellular metabolic activity.

### 4.5. Assessment of Mitochondrial Membrane Potential in C. albicans

To evaluate mitochondrial function, *C. albicans* cells (1 × 10^5^ cells/mL) were cultured in RPMI medium and treated with compounds ATF19, ATF20, or MB22 at 1× MIC for 24 h at 37 °C in transparent 96-well plates. Mitochondrial membrane potential was assessed using the JC-10 Mitochondrial Membrane Potential Assay Kit (Abcam, Cambridge, United Kingdom), according to the manufacturer’s instructions [41].

This assay is based on the potential-dependent accumulation of JC-10 dye within mitochondria, where intact mitochondria promote dye aggregation, while depolarized mitochondria favor the monomeric form. Fluorescence signals were measured at 490–525 nm (monomeric form) and 540–590 nm (aggregated form). The ratio of monomeric to aggregated fluorescence was calculated to quantify mitochondrial membrane integrity.

### 4.6. Effect of Compounds on C. albicans Filamentation

Yeast cells were suspended at a concentration of 1 × 10^4^ cells/mL in RPMI medium supplemented with 10% FBS and incubated in transparent 96-well plates at 37 °C [42]. Cells were treated with compounds ATF19, ATF20, or MB22 at 1× MIC and incubated for 2 h to induce filamentation. Following incubation, treated and untreated *C. albicans* cells were examined using light microscopy. The proportion of filamentous (hyphal) forms was quantified by counting cells in three randomly selected microscopic fields per well.

### 4.7. Quantification of Cell Wall Mannans

The *C. albicans* cell wall is composed primarily of chitin, β-glucans, and mannans. Alterations in mannan content were assessed as an indicator of cell wall integrity. *C. albicans* cells (1 × 10^5^ cells/mL) were treated with compounds ATF19, ATF20, or MB22 at 1× MIC in 200 µL RPMI medium and incubated for 24 h in black 96-well plates. Cells were then collected by centrifugation and stained with Galanthus nivalis lectin (GNL; Vector Laboratories, Burlingame, CA, USA) for 1 h [42].

After washing, 100 µL of PBS was added to each well, and fluorescence was measured at 450 nm to quantify mannan levels. GNL-stained cells were also visualized by fluorescence microscopy using a Zeiss AxioImager system at the Lille Cell Imaging Platform (BiCels).

### 4.8. Biofilm Formation Assay

*C. albicans* cells (5 × 10^3^ cells/mL) were cultured in RPMI medium supplemented with 20% FBS at 37 °C for 6 h to allow initial adhesion. Cells were subsequently treated with compounds ATF19, ATF20, or MB22 at 1× MIC and incubated for an additional 24 h in transparent 96-well plates. Wells were gently washed three times with PBS to remove non-adherent cells [40]. Biofilms were stained with crystal violet for 30 min (Honeywell Fluka, Sevrey, France), followed by ethanol destaining to remove excess dye. Biofilm biomass was quantified by measuring absorbance at 550 nm. Representative biofilm images were acquired using a Leica DMI8 microscope at the Lille imaging facility.

### 4.9. C. elegans Infection Model

*C. albicans* cells were cultured in liquid Sabouraud medium at 37 °C for 24 h. The wild-type N2 strain of *C. elegans* was maintained on nematode growth medium seeded with *Escherichia coli* OP50 at 20 °C. Worm populations were synchronized prior to infection [20,42].

*C. albicans* cells were plated on Brain Heart Infusion (BHI) agar supplemented with amikacin (45 µg/mL; Mylan, Paris, France) for 30 min. Synchronized nematodes were washed extensively with M9 buffer containing 90 µg/mL amikacin to remove *E. coli* and promote ingestion of *C. albicans*. Worms were then exposed to *C. albicans* on BHI plates for 6 h at 20 °C.

Following infection, nematodes were collected, counted, and distributed (10–20 worms per well) into transparent 12-well plates containing M9 buffer supplemented with 5% BHI and treated with compounds ATF19, ATF20, or MB22 at 1× MIC. Worm survival was monitored daily for 5 days post-infection. At the end of the experiment, representative images were acquired using a Leica DMI8 microscope at the Lille Cell Imaging Platform.

### 4.10. Statistical Analysis

All statistical analyses were performed using GraphPad Prism software (GraphPad Software, version 11.0.0, La Jolla, CA, USA). Differences between experimental groups were evaluated using the non-parametric Mann–Whitney–Wilcoxon test. Statistical significance was defined as *p* < 0.05, with additional thresholds at *p* < 0.01 and *p* < 0.001.

## 5. Conclusions

In conclusion, this study demonstrates that the chemical hybridization of natural product scaffolds is an effective strategy for generating antifungal agents with enhanced potency, antivirulence activity, and low host toxicity. Among the compounds investigated, the ferulic acid–citronellol hybrid ATF20, the sinapic derivative ATF19, and the propylate MB22 emerge as promising leads. Their multifaceted mode of action, which combines growth inhibition, virulence suppression, and host protection, makes them attractive candidates for further preclinical development and mechanistic exploration.

## Figures and Tables

**Table 1:** Structures, log*P*, and MIC of screened hybrid biosourced molecules based on ferulic acid.

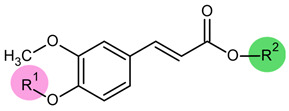								
Entry	Code	R^1^	R^2^	M (g/mol)	*^a^*log*P*	MIC (µg/mL)	MIC (µM)	SD
1	F0	H	H	194.18	1.36	97.09	500.0	±0.093
2	F1	COCH_3_	H	236.22	1.76	118.11	500.0	±0.019
3	F2	COCH_2_CH_3_	H	250.24	2.13	12.51	50.0	±0.093
4	MB22	CO(CH_2_)_2_CH_3_	H	264.27	2.46	2.64	10.0	±0.087
5	F4	CO (CH_2_)_3_CH_3_	H	278.30	2.78	139.15	500.0	±0.030
6	F5	CO (CH_2_)_4_CH_3_	H	292.32	3.18	9.83	33.6	±0.073
7	F6	CO (CH_2_)_5_CH_3_	H	306.35	3.53	15.3	50.0	±0.16
8	F7	CO (CH_2_)_6_CH_3_	H	320.38	3.86	80.09	250.0	±0.014
9	F8	CO (CH_2_)_7_CH_3_	H	334.42	4.28	16.72	50.0	±0.061
10	F9	H	CH_3_	208.21	1.76	52.0525	250.0	±0.0083
11	F10	H	CH_2_CH_3_	222.23	2.11	55.557	250.0	±0.012
12	F11	H	(CH_2_)_2_CH_3_	236.26	2.45	118.13	500.0	±0.011
13	F12	H	(CH_2_)_3_CH_3_	250.29	2.80	62.57	250.0	±0.070
14	F13	H	(CH_2_)_4_CH_3_	264.31	3.16	66.07	250.0	±0.038
15	F14	H	(CH_2_)_5_CH_3_	278.34	3.53	13.9	50.0	±0.14
16	F15	H	(CH_2_)_6_CH_3_	292.37	3.83	14.6	50.0	±0.11
17	F16	H	(CH_2_)_9_CH_3_	334.39	4.26	15.3	46.0	±0.10
18	F17	H	(CH_2_)_2_CH(CH_3_)_2_	264.31	3.06	66.07	250.0	±0.033
19	F18	H	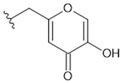	318.27	4.16	90.07	283.0	±0.012
20	F19	H	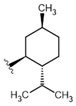	318.28	1.57	>150	>500	-
21	ATF20	H	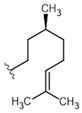	332.43	4.43	16.6	50.0	±0.13
22	F21	CH_3_	CH_3_	250.24	2.20	125.12	500.0	±0.033
23	CI	Citronellol		156.27	2.92	78.13	500.0	±0.093
24	KA	Kojic acid		142.11	−0.16	90.0775	634.0	±0.027
25	ME	(-)Menthol		156.26	2.58	78.101	500.0	±0.0087
26	SA	Sinapic acid		224.21	1.32	112.105	500.0	±0.0062
27	IE	*Iso*-eugenol		164.23	2.43	82.15	500.2	±0.01
28	ATF19	* 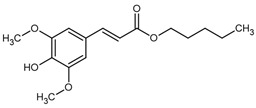 *		294.34	3.11-	14.71	50.0	±0.060
29	E2	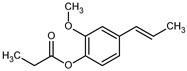		220.26	2.98	10.3115	46.8	±0.11

^a^Log *P* = Partition coefficient representing the ratio of concentrations between octanol and water (source: SwissADME).

## Data Availability

The datasets used and/or analyzed during the current study are available from the corresponding author on reasonable request.

## Supplementary Materials

The following supporting information can be downloaded at https://www.mdpi.com/article/10.3390/ijms27062859/s1. References [43,44,45,46,47,48,49,50,51,52] are cited in the Supplementary Materials.
