# Supplementary material for: Nature-Derived Ferulic Acid Hybrids with Enhanced Antifungal and Antivirulence Activity Against Candida albicans
Source: Int J Mol Sci. 2026 Mar 21;27(6):2859. doi: 10.3390/ijms27062859 (PMC13026692; doi:10.3390/ijms27062859)
Supplement: Supplementary file 1 [file ijms-27-02859-s001.zip › ijms-4187002-supplementary.pdf]

SUPPORTING INFORMATION

Nature-Derived Ferulic Acid Hybrids with Enhanced Antifungal and Antivirulence Activity against *Candida albicans*

Dylan Lambert, Celia Lemaire, Muriel Billamboz, Samir Jawhara

---

Table of contents

I. Generalities

II. Synthetic routes and characterization of trans-ferulic acid derivatives

1. Modification of R1

2. Modification of R2

III. 1H and 13C NMR spectra of the compounds

IV. Quantification of ROS production in *C. albicans*

V. References

I. Generalities.

All commercial products and solvents were used without prior purification. The syntheses were monitored by thin-layer chromatography and NMR. Thin-layer chromatography was performed on Macherey Nagel silica gel plates with a fluorescent indicator and developed under a UV lamp at 254 nm. NMR spectra were acquired at 400 MHz for ^{1}H NMR and at 100 MHz for ^{13}C NMR on a Varian MR 400 spectrometer. Chemical shifts (δ) are given in parts per million (ppm) relative to the internal standard TMS at 25°C. Peaks are annotated as follows: singlet (s), broad singlet (bs), doublet (d), doublet of doublets (dd), triplet (t), quadruplet (q), doublet of quadruplets (qd), quintuplet (quint.), sextuplet (sext.) and multiplet (m). The coupling constants *J* are given in Hertz (Hz). Column chromatography was performed using a CombiFlash Rf Companion (Teledyne-Isco System) and RediSep pre-packed columns. Infrared spectra were obtained using a Varian 640-IR FT-IR spectrometer. Melting points were measured using an OptiMelt® MPA 100 device.

II. Synthetic routes and characterization of *trans*-ferulic acid derivatives

1. Modification of R^1

The carboxylic acid (5.15 mmol) is dissolved in 20 mL of dichloromethane under magnetic stirring and immersed in an ice bath. When the mixture reaches 0-5°C, 1-ethyl-3-(3-dimethylaminopropyl)carbodiimide hydrochloride (5.66 mmol, 1.1 eq) and 4-dimethylaminopyridine (1.03 mmol, 0.2 eq) were added to activate the carboxylic acid. *Trans*-

ferulic acid (5.15 mmol, 1 eq) was then added to the reaction media. The progress of the esterification was monitored by TLC. After complete conversion of the *trans*-ferulic acid, without prior washing, the crude reaction product was purified by flash chromatography using a heptane/ethyl acetate gradient.

F1. (2*E*)-3-[4-(acetyloxy)-3-methoxyphenyl]prop-2-enoic acid

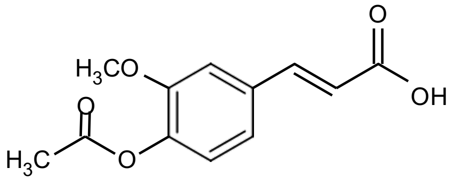

Yield : 8 %

Aspect : white solid

Melting point: 182°C

^{1}H NMR (400 MHz, DMSO) δ ppm : 12.38 (bs, 1H. OH) ; 7.57 (d, 1H, CH, *J*=16.0 Hz) ; 7.48 (d, 1H, CH, *J*=2.1 Hz) ; 7.26 (dd, 1H, CH, *J*=1.7/8.2 Hz) ; 7.12 (d, 1H, CH, *J*=8.3 Hz) ; 6.58 (d, 1H, CH, *J*=16.1 Hz) ; 3.82 (s, 3H, CH_3) ; 2.26 (s, 3H, CH_3)

^{13}C NMR (100 MHz, DMSO) δ ppm : 168.3 (CO) ; 167.5 (CO) ; 151.0 (CO) ; 143.2 (CH) ; 140.7 (CO) ; 133.1 (C) ; 123.0 (CH) ; 121.2 (CH) ; 119.4 (CH) ; 111.7 (CH) ; 55.9 (CH_3) ; 20.3 (CH_3)

F2. (2*E*)-3-[3-methoxy-4-(propanoyloxy)phenyl]prop-2-enoic acid

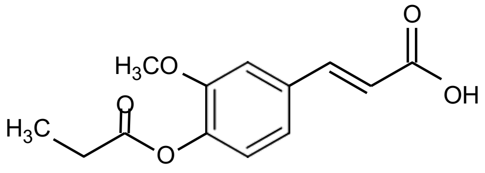

Yield : 8 %

Aspect : solide blanc

Melting point: 152 °C

^{1}H NMR (400 MHz, DMSO) δ ppm : 12.36 (bs, 1H, OH) ; 7.57 (d, 1H, CH, J=15.8 Hz) ; 7.48 (d, 1H, CH, J=1.8 Hz) ; 7.26 (dd, 1H, CH, J=1.5/8.2 Hz) ; 7.12 (d, 1H, CH, J=8.3 Hz) ; 6.59 (d, 1H, CH, J=16.0 Hz) ; 3.82 (s, 3H, CH_3) ; 2.59 (q, 2H, CH_2, J=7.38 Hz) ; 1.14 (t, 3H, CH_3, J=7,5 Hz)

^{13}C NMR (100 MHz, DMSO) δ ppm : 172.2 (CO) ; 168.0 (CO) ; 151.6 (CO) ; 143.8 (CH) ; 141.3 (CO) ; 133.6 (C) ; 123.6 (CH) ; 121.7 (CH) ; 119.9 (CH) ; 112.2 (CH) ; 56.4 (CH_3) ; 27.0 (CH_2) ; 9.4 (CH_3)

MB22. (2*E*)-3-[4-(butanoyloxy)-3-methoxyphenyl]prop-2-enoic acid

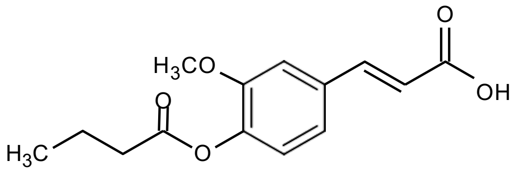

Yield : 25 %

Aspect : solide blanc

Melting point: 151°C

^{1}H NMR (400 MHz, DMSO) δ ppm : 12.38 (bs, 1H, OH) ; 7.57 (d, 1H, CH, J=15.9 Hz) ; 7.48 (d, 1H, CH, J=1.6 Hz) ; 7.26 (dd, 1H, CH, J=1.5/8.2 Hz) ; 7.10 (d, 1H, CH, J=8.3 Hz) ; 6.58 (d, 1H, CH, J=16.0 Hz) ; 3.81 (s, 3H, CH_3) ; 2.54 (t, 2H, CH_2, J=7.4 Hz) ; 1.66 (sext., 2H, CH_2, J=7.5 Hz) ; 0.98 (t, 3H, CH_3, J=7.5 Hz)

^{13}C NMR (100 MHz, DMSO) δ ppm : 170.8 (CO) ; 167.5 (CO) ; 151.0 (CO) ; 143.2 (CH) ; 140.7 (CO) ; 133.1 (C) ; 123.0 (CH) ; 121.2 (CH) ; 119.4 (CH) ; 111.7 (CH) ; 55.9 (CH_3) ; 34.9 (CH_2) ; 17.9 (CH_2) ; 13.1 (CH_3)

F4. (2*E*)-3-[3-methoxy-4-(pentanoyloxy)phenyl]prop-2-enoic acid

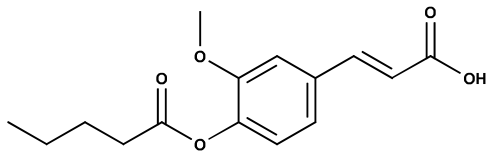

Yield : 26 %

Aspect : solide blanc

Melting point: 116°C

^{1}H NMR (400 MHz, CDCl3) δ ppm : 7.81 (d, 1H, CH, J=15.7 Hz) ; 7.18 (dd, 1H, CH, J=1.6/7.8 Hz) ; 7.14 (d, 1H, CH, J=1.6 Hz) ; 7.08 (d, 1H, CH, J=8.0 Hz) ; 6.47 (d, 1H, CH, J=15.9 Hz) ; 3.87 (s, 3H, CH_3); 2.60 (t, 2H, CH_2, J=7.2 Hz) ; 1.76 (quint., 2H, CH_2, J=7.3 Hz) ; 1.47 (sext., 2H, CH_2, J=7.6 Hz) ; 0.98 (t, 3H, CH_3, J=7.3 Hz)

^{13}C NMR (100 MHz, DMSO) δ ppm : 170.9 (CO) ; 162.6 (CO) ; 151.1 (CO) ; 147.9 (CH) ; 141.7 (CO) ; 132.4 (C) ; 123.2 (CH) ; 122.4 (CH) ; 116.9 (CH) ; 112.4 (CH) ; 56.0 (CH_3) ; 32.8 (CH_2) ; 26.4 (CH_2) ; 21.3 (CH_2) ; 13.5 (CH_3)

F5. (2*E*)-3-[4-(hexanoyloxy)-3-methoxyphenyl]prop-2-enoic acid

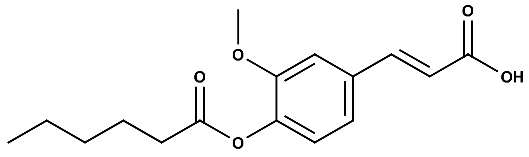

Yield : 26 %

Aspect : solide blanc

Melting point: 142°C

^{1}H NMR (400 MHz, CDCl3) δ ppm : 12.34 (bs, 1H, OH) ; 7.54 (d, 1H, CH, J=16.0 Hz) ; 7.44 (d, 1H, CH, J=2.0 Hz) ; 7.22 (dd, 1H, CH, J=1.9/8.2 Hz) ; 7.07 (d, 1H, CH, J=8.2 Hz) ; 6.55 (d, 1H, CH, J=16.0 Hz) ; 3.78 (s, 3H, CH_3) ; 2.52 (t, 2H, CH_2, J=7.4 Hz) ; 1.61 (quint., 2H, CH_2, J=7.9 Hz) ; 1.34-1.27 (m, 4H, 2xCH_2) ; 0.86 (t, 3H, CH_3, J=7.9 Hz)

^{13}C NMR (100 MHz, CDCl3) δ ppm : 171.4 (CO) ; 168.0 (CO) ; 151.6 (CO) ; 143.8 (CH) ; 141.3 (CO) ; 133.6 (C) ; 123.6 (CH) ; 121.8 (CH) ; 119.9 (CH) ; 112.3 (CH) ; 56.4 (CH_3) ; 33.6 (CH_2) ; 30.9 (CH_2) ; 24.6 (CH_2) ; 22.2 (CH_2) ; 14.3 (CH_3)

F6. (2*E*)-3-[4-(heptanoyloxy)-3-methoxyphenyl]prop-2-enoic acid

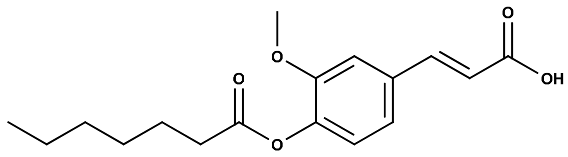

Yield : 27 %

Aspect : solide blanc

Melting point: 136°C

^{1}H NMR (400 MHz, DMSO) δ ppm : 12.36 (bs, 1H, OH) ; 7.54 (d, 1H, CH, J=15.7 Hz) ; 7.44 (d, 1H, CH, J=1.2 Hz) ; 7.23 (dd, 1H, CH, J=1.6/8.2 Hz) ; 7.06 (d, 1H, CH, J=8.3 Hz) ; 6.55 (d, 1H, CH, J=16.0 Hz) ; 3.78 (s, 3H, CH_3) ; 2.52 (t, 2H, CH_2, J=6.9 Hz) ; 1.60 (quint., 2H, CH_2, J=7.4 Hz) ; 1.41-1.29 (m, 6H, 3xCH_2) ; 0.85 (t, 3H, CH_3, J=7.1 Hz)

^{13}C NMR (100 MHz, DMSO) δ ppm : 170.9 (CO) ; 167.4 (CO) ; 151.0 (CO) ; 143.2 (CH) ; 140.7 (CO) ; 133.1 (C) ; 123.0 (CH) ; 121.2 (CH) ; 119.4 (CH) ; 111.7 (CH) ; 55.8 (CH_3) ; 33.0 (CH_2) ; 30.7 (CH_2) ; 27.8 (CH_2) ; 24.3 (CH_2) ; 21.8 (CH_2) ; 13.8 (CH_3)

F7. (2*E*)-3-[3-methoxy-4-(octanoyloxy)phenyl]prop-2-enoic acid

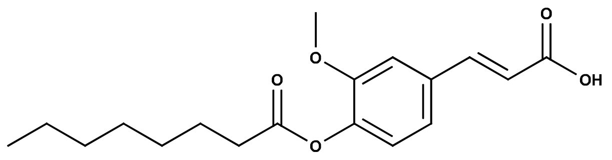

Yield : 16 %

Aspect : solide blanc

Melting point: 144°C

^{1}H NMR (400 MHz, DMSO) δ ppm : 12.36 (bs, 1H, OH) ; 7.54 (d, 1H, CH, J=15.7 Hz) ; 7.44 (d, 1H, CH, J=2.0 Hz) ; 7,22 (dd, 1H, CH, J=2.0/8.2 Hz) ; 7.06 (d, 1H, CH, J=8.2 Hz) ; 6.55 (d, 1H, CH, J=16.0 Hz) ; 3.78 (s, 3H, CH_3) ; 2.52 (t, 2H, CH_2, J=7.0 Hz) ; 1.60 (quint., 2H, CH_2, J=7.4 Hz) ; 1.35-1.22 (m, 8H, 4xCH_2) ; 0.84 (t, 3H, CH_3, J=6.6 Hz)

^{13}C NMR (100 MHz, DMSO) δ ppm : 170.9 (CO) ; 167.5 (CO) ; 151.0 (CO) ; 143.2 (CH) ; 140.8 (CO) ; 133.1 (C) ; 123.0 (CH) ; 121.3 (CH) ; 119.4 (CH) ; 111.7 (CH) ; 55.9 (CH_3) ; 33.0 (CH_2) ; 31.0 (CH_2) ; 28.2 (CH_2) ; 28.1 (CH_2) ; 24.4 (CH_2) ; 21.9 (CH_2) ; 13.8 (CH_3)

F8. (2*E*)-3-[3-methoxy-4-(nonanoyloxy)phenyl]prop-2-enoic acid

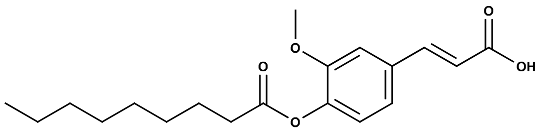

Yield : 25 %

Aspect : white solid

Melting point: 122°C

^{1}H NMR (400 MHz, DMSO) δ ppm : 12.38 (bs, 1H, OH) ; 7.57 (d, 1H, CH, J=16.0 Hz) ; 7.48 (d, 1H, CH, J=1.5 Hz) ; 7.26 (dd, 1H, CH, J=2.0/8.1 Hz) ; 7.09 (d, 1H, CH, J=8.2 Hz) ; 6.58 (d, 1H, CH, J=16.0 Hz) ; 3.81 (s, 3H, CH_3) ; 2.55 (t, 2H, CH_2, J=7.0 Hz) ; 1.63 (quint., 2H, CH_2, J=7.4 Hz) ; 1.38-1.24 (m, 10H, 5xCH_2) ; 0.87 (t, 3H, CH_3, J=7.4 Hz)

2. Modification of R^2

Synthesis of compounds F9-10

Trans-ferulic acid (5.15 mmol) and *para*-toluene sulfonic acid (0.51 mmol, 0.1 eq) were dissolved in 15 mL (excess) of alcohol (methanol for compound F9 and ethanol for compound F10). The reaction mixture was placed under magnetic stirring and reflux. The progress of esterification was monitored by TLC. After complete conversion of *trans*-ferulic acid to ferulate, the solvent was evaporated. The crude reaction mixture was then solubilized in 30 mL of ethyl acetate and washed with a saturated $NaHCO_3$ solution (3x15 mL) followed by a saturated NaCl solution (3x15 mL). The organic phase was dried using anhydrous magnesium sulfate, filtered, and concentrated under vacuum. If necessary, the crude product was purified by flash chromatography using a heptane/ethyl acetate gradient.

F9. Methyl (2*E*)-3-(4-hydroxy-3-methoxyphenyl)prop-2-enoate [i,ii]

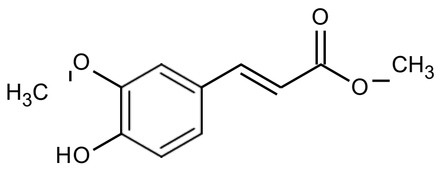

Yield : 95 %

Aspect : brown oil

TLC Rf : 0.45 (*n*-heptane/ethyle acetate : 50/50)

^{1}H NMR (400 MHz, $CDCl_3$) δ ppm : 7.62 (d, 1H, CH, J = 15.5 Hz) ; 7.07 (dd, 1H, CH, J = 2.2/8.4 Hz) ; 7.02 (d, 1H, CH, J = 2.0 Hz) ; 6.91 (d, 1H, CH, J = 8.3 Hz) ; 6.29 (d, 1H, CH, J = 16.0 Hz) ; 5.95 (bs, 1H, OH) ; 3.92 (s, 3H, CH_3) ; 3.79 (s, 3H, CH_3)

^{13}C NMR (100 MHz, $CDCl_3$) δ ppm : 167.7 (CO) ; 148.0 (CO) ; 146.8 (CO) ; 144.9 (CH) ; 126.9 (C) ; 123.0 (CH) ; 115.1 (CH) ; 114.7 (CH) ; 109.4 (CH) ; 55.9 (CH_3) ; 51.6 (CH_3)

F10. Ethyl (2*E*)-3-(4-hydroxy-3-methoxyphenyl)prop-2-enoate [i,iii]

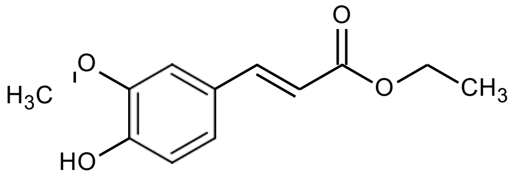

Yield : 16 %

Aspect : brown oil

TLC Rf : 0.52 (*n*-heptane/ethyle acetate : 50/50)

^{1}H NMR (400 MHz, $CDCl_3$) δ ppm : 7.58 (d, 1H, CH, *J* = 16.4 Hz) ; 7.05 (dd, 1H, CH, *J* = 1.6/8.0 Hz) ; 7.00 (d, 1H, CH, *J* = 1.6 Hz) ; 6.89 (d, 1H, CH, *J* = 8.4 Hz) ; 6.26 (d, 1H, CH, *J* = 16.0 Hz) ; 5.89 (bs, 1H, OH) ; 4.23 (q, 2H, CH_2, *J* = 7.6 Hz) ; 3.90 (s, 3H, CH_3) ; 1.31 (t, 3H, CH_3, *J* = 7.6 Hz)

^{13}C NMR (100 MHz, $CDCl_3$) δ ppm : 167.3 (CO) ; 147.9 (CO) ; 146.7 (CO) ; 144.6 (CH) ; 127.0 (C) ; 123.0 (CH) ; 115.6 (CH) ; 114.7 (CH) ; 109.3 (CH) ; 60.3 (CH_2) ; 55.9 (CH_3) ; 14.3 (CH_3)

Synthesis of compounds F11-17 and ATF20

Trans-ferulic acid (5.15 mmol) and *para*-toluene sulfonic acid (0.51 mmol. 0.1 eq) were dissolved in 15 mL of toluene, then alcohol (5.15 mmol, 1 eq) was added. The reaction mixture was placed under magnetic stirring and refluxed. The progress of esterification was monitored by TLC. Aftercomplete conversion of *trans*-ferulic acid to ferulate, the solvent was evaporated. The crude reaction mixture was then solubilized in 30 mL of ethyl acetate and washed with a saturated $NaHCO_3$ solution (3x15 mL) followed by a saturated NaCl solution (3x15 mL). The organic phase was dried using anhydrous magnesium sulfate, filtered, and concentrated under vacuum. If necessary, the crude product was purified by flash chromatography using a heptane/ethyl acetate gradient.

F11. Propyl (2*E*)-3-(4-hydroxy-3-methoxyphenyl)prop-2-enoate [i,iv]

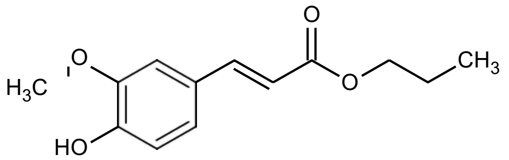

Yield : 18 %

Aspect : colourless oil

TLC Rf : 0.55 (*n*-heptane/ethyle acetate : 50/50)

^{1}H NMR (400 MHz, $CDCl_3$) δ ppm : 7.61 (d, 1H, CH, *J* = 16.4 Hz) ; 7.08 (dd, 1H, CH, *J* = 2.0/7.6 Hz) ; 7.03 (d, 1H, CH, *J* = 2.0 Hz) ; 6.92 (d, 1H, CH, *J* = 8.0 Hz) ; 6.30 (d, 1H, CH, *J* = 15.6 Hz) ; 5.86 (bs, 1H, OH) ; 4.16 (t, 2H, CH_2, *J* = 7.2 Hz) ; 3.93 (s, 3H, CH_3) ; 1.71 (sext., 2H, CH_2, *J* = 7.2 Hz); 0.99 (t, 3H, CH_3, *J* = 7.2 Hz)

^{13}C NMR (100 MHz, $CDCl_3$) δ ppm : 167.4 (CO) ; 147.9 (CO) ; 146.8 (CO) ; 144.6 (CH) ; 127.1 (C) ; 123.0 (CH) ; 115.7 (CH) ; 114.7 (CH) ; 109.3 (CH) ; 66.0 (CH_2) ; 55.9 (CH_3) ; 22.1 (CH_2) ; 10.5 (CH_3)

F12. Butyl (2*E*)-3-(4-hydroxy-3-methoxyphenyl)prop-2-enoate [i,iv]

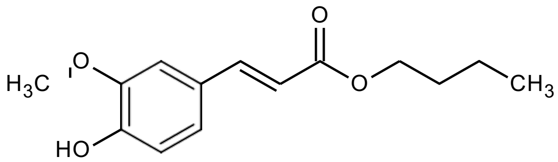

Yield : 42 %

Aspect : colourless oil

TLC Rf : 0.55 (*n*-heptane/ ethyle acetate : 50/50)

^{1}H NMR (400 MHz, CDCl3) δ ppm : 7.61 (d,1H, CH, J=15.7 Hz) ; 7.07 (dd, 1H, CH, J=1.5/8.1 Hz) ; 7.03 (d, 1H, CH, J=2.0 Hz) ; 6.91 (d, 1H, CH, J=8.2 Hz) ; 6.29 (d, 1H, CH, J=16.0 Hz); 5.89 (s, 1H, OH) ; 4.20 (t, 2H, CH_2, J=6.7 Hz) ; 3.92 (s, 3H, CH_3) ; 1.69 (quint., 2H, CH_2, J=6.8 /13.3 Hz) ; 1.44 (sext., 2H, CH_2, J=7.4 Hz) ; 0.96 (t, 3H, CH_3, J=7.4 Hz)

^{13}C NMR (100 MHz, CDCl3) δ ppm : 167.5 (CO) ; 148.0 (CO) ; 146.9 (CO) ; 144.7 (CH) ; 127.2 (C) ; 123.2 (CH) ; 115.8 (CH) ; 114.8 (CH) ; 109.4 (CH) ; 64.4 (CH_2) ; 56.0 (CH_3) ; 30.9 (CH_2) ; 19.3 (CH_2) ; 13.9 (CH_3)

F13. Pentyl (2*E*)-3-(4-hydroxy-3-methoxyphenyl)prop-2-enoate [i]

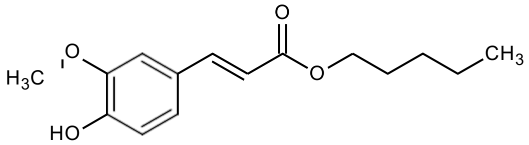

Yield : 63 %

Aspect : colourless oil

TLC Rf : 0.57 (*n*-heptane/ ethyle acetate : 50/50)

^{1}H NMR (400 MHz, CDCl3) δ ppm : 7.61 (d,1H, CH, J=16.1 Hz) ; 7.07 (dd, 1H, CH, J=1.5/8.2 Hz) ; 7.03 (d, 1H, CH, J=2.1 Hz) ; 6.92 (d, 1H, CH, J=8.0 Hz) ; 6.29 (d, 1H, CH, J=16.0 Hz); 5.85 (s, 1H, OH) ; 4.19 (t, 2H, CH_2, J=6.6 Hz) ; 3.92 (s, 3H, CH_3) ; 1.71 (m, 2H, CH_2) ; 1.38 (m, 4H, 2xCH_2) ; 0.93 (t, 3H, CH_3, J=6.7 Hz)

^{13}C NMR (100 MHz, CDCl3) δ ppm : 167.4 (CO) ; 147.9 (CO) ; 146.7 (CO) ; 144.6 (CH) ; 127.1 (C) ; 123.0 (CH) ; 115.7 (CH) ; 114.7 (CH) ; 109.3 (CH) ; 64.6 (CH_2) ; 55.9 (CH_3) ; 28.5 (CH_2) ; 28.1 (CH_2) ; 22.4 (CH_2) ; 14.0 (CH_3)

F14. Hexyl (2*E*)-3-(4-hydroxy-3-methoxyphenyl)prop-2-enoate [v]

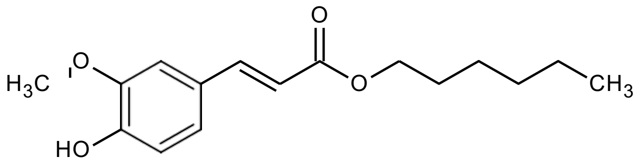

Yield : 55 %

Aspect : colourless oil

TLC Rf : 0.58 (n-heptane/ ethyle acetate : 50/50)

1H NMR (400 MHz, CDCl3) δ ppm : 7.61(d,1H, CH, J=16.1 Hz) ; 7.07 (dd, 1H, CH, J=1.9 Hz/8.2 Hz) ; 7.03 (d, 1H, CH, J=1.9 Hz) ; 6.91 (d, 1H, CH, J=8.3 Hz) ; 6.29 (d, 1H, CH, J=16.1 Hz) ; 5.88 (bs, 1H, OH) ; 4.19 (t, 2H, CH_2, J=6.9 Hz) ; 3.92 (s, 3H, CH_3) ; 1.71-1.66 (m, 2H, CH_2) ; 1.42-1.30 (m, 6H, $3xCH_2$) ; 0.90 (t, 3H, CH_3, J=7.3 Hz)

13C NMR (100 MHz, CDCl3) δ ppm : 167.5 (CO) ; 148.0 (CO) ; 146.9 (CO) ; 144.7 (CH) ; 127.2 (C) ; 123.2 (CH) ; 115.8 (CH) ; 114.8 (CH) ; 109.4 (CH) ; 64.7 (CH_2) ; 56.0 (CH_3) ; 31.6 (CH_2) ; 28.9 (CH_2) ; 25.8 (CH_2) ; 22.7 (CH_2) ; 14.1 (CH_3)

F15. Heptyl (2E)-3-(4-hydroxy-3-méthoxyphényl)prop-2-enoate [vi]

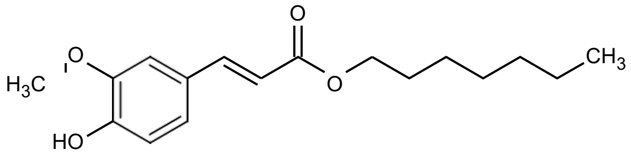

Yield : 59 %

Aspect : huile incolore

TLC Rf : 0.59 (n-heptane/ ethyle acetate : 50/50)

^{1}H NMR (400 MHz, $CDCl_3$) δ ppm : 7.61(d,1H, CH, J=16.0 Hz) ; 7.07 (dd, 1H, CH, J=2.0/8.0 Hz) ; 7.03 (d, 1H, CH, J=1.6 Hz) ; 6.91 (d, 1H, CH, J=8.4 Hz) ; 6.29 (d, 1H, CH, J=16.0 Hz); 5.85 (s, 1H, OH) ; 4.19 (t, 2H, CH_2, J=6.8 Hz) ; 3.93 (s, 3H, CH_3) ; 1.70 (quint., 2H, CH_2, J=6.4 Hz) ; 1.40-1.29 (m, 8H, $4xCH_2$) ; 0.89 (t, 3H, CH_3, J=6.8 Hz)

^{13}C NMR (100 MHz, $CDCl_3$) δ ppm : 167.4 (CO) ; 147.9 (CO) ; 146.7 (CO) ; 144.6 (CH) ; 127.0 (C) ; 123.0 (CH) ; 115.7 (CH) ; 114.7 (CH) ; 109.3 (CH) ; 64.6 (CH_2) ; 55.9 (CH_3) ; 31.7 (CH_2) ; 28.9 (CH_2) ; 28.7 (CH_2) ; 25.9 (CH_2) ; 22.6 (CH_2) ; 14.1 (CH_3)

F16. Octyl (2E)-3-(4-hydroxy-3-méthoxyphényl)prop-2-enoate [vii]

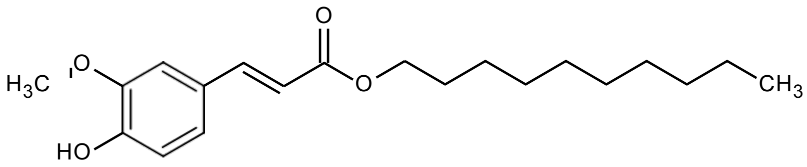

Yield : 60 %

Aspect : colourless oil

TLC Rf : 0.61 (*n*-heptane/ ethyle acetate : 50/50)

^{1}H NMR (400 MHz, $CDCl_3$) δ ppm : 7.58 (d, 1H, CH, J= 15.6 Hz) ; 7.04 (dd, 1H, CH, J=1.6/8.0 Hz) ; 7.00 (d, 1H, CH, J=2.0 Hz) ; 6.89 (d, 1H, CH, J=8.4 Hz) ; 6.26 (d, 1H, CH, J=16.0 Hz) ; 5.98 (s, 1H, OH) ; 4.16 (t, 2H, CH_2, J=6.8 Hz) ; 3.89 (s, 3H, CH_3) ; 1.67 (quint., 2H, CH_2, J=6.4 Hz) ; 1.40-1.23 (m, 14H, 7xCH_2) ; 0.85 (t, 3H, CH_3, J=6.8 Hz)

^{13}C NMR (100 MHz, $CDCl_3$) δ ppm : 167.4 (CO) ; 147.9 (CO) ; 146.7 (CO) ; 144.6 (CH) ; 127.0 (C) ; 123.0 (CH) ; 115.7 (CH) ; 114.7 (CH) ; 109.3 (CH) ; 64.6 (CH2) ; 55.9 (CH_3) ; 31.9 (CH_2) ; 29.5 (2xCH_2) ; 29.3 (2xCH_2) ; 28.8 (CH_2) ; 26.0 (CH_2) ; 22.7 (CH_2) ; 14.1 (CH_3)

F17. Isopentyl (E)-3-(4-hydroxy-3-méthoxyphényl)prop-2-enoate [i]

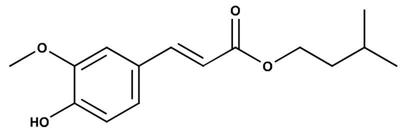

Yield : 12 %

Aspect : huile incolore

^{1}H NMR (400 MHz, $CDCl_3$) δ ppm : 7.60 (d, 1H, CH, J = 16.0 Hz) ; 7.07 (dd, 1H, CH, J = 1.9/8.2 Hz) ; 7.03 (d, 1H, CH, J = 1.9 Hz) ; 6.91 (d, 1H, CH, J = 8.3 Hz) ; 6.28 (d, 1H, CH, J = 16.0 Hz) ; 5.86 (s, 1H, OH) ; 4.23 (t, 2H, CH_2, J=6.8 Hz) ; 3.93 (s, 3H, CH_3) ; 1.75 (sept., 1H, CH, J=6.5 Hz) ; 1.59 (q, 2H, CH_2, J=6.5 Hz) ; 0.96 (d, 6H, 2xCH_3, J=6.8 Hz)

F18. (5-hydroxy-4-oxo-4H-pyran-2-yl)methyl(E)-3-(4-hydroxy-3-methoxyphenyl) acrylate [viii]

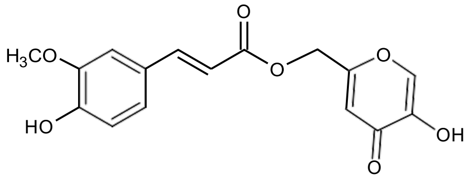

Trans-ferulic acid (5.15 mmol) was dissolved in a flask containing 40 mL of methanol in the presence of sodium hydroxide (5.15 mmol, 1 eq). The reaction mixture thus formed was kept under magnetic stirring at room temperature for 2 hours. The methanol was evaporated under vacuum, then chlorokojic acid (5.15 mmol, 1 eq) was added to the flask along with 70 mL of dimethylformamide. The flask, equipped with a condenser, was heated to 110°C for 6 hours. The crude reaction mixture was then evaporated to remove the solvent and resolved in 100 mL of ethyl acetate. The organic phase was washed successively with a 5% m/v hydrochloric acid solution (3 x 50 mL) and distilled water (3 x 50 mL), then dried using anhydrous magnesium sulfate, filtered, purified by adding a few milligrams of vegetable charcoal (Norit – 10-74 µm) removed by filtration, and then evaporated under vacuum. Traces of residual impurities were removed by washing the solid with dichloromethane, a solvent in which it is insoluble. The reaction product is a beige-colored solid obtained with a yield of 38%.

Yield : 38 %
Aspect : off-white solid
Melting point: 182°C
^{1}H NMR (400 MHz, $CDCl_3$) δ ppm : 9.66 (s, 1H, OH) ; 9.24 (s, 1H, OH) ; 8.11 (s, 1H, CH) ; 7.63 (d, 1H, CH, J=15.6 Hz) ; 7.37 (d, 1H, CH, J=1.5 Hz) ; 7.16 (dd, 1H, CH, J=1.5/7.7 Hz) ; 6.80 (d, 1H, CH, J=8.2 Hz) ; 6.58 (d, 1H, CH, J=16.0 Hz) ; 6.52 (s, 1H, CH) ; 5.07 (s, 2H, CH_2); 3.82 (s, 3H, CH_3)
^{13}C NMR (100 MHz, $CDCl_3$) δ ppm : 173.5 (CO) ; 165. 8 (CO) ; 161.7 (C) ; 149.5 (CO) ; 147.8 (CO) ; 146.3 (CO) ; 145.9 (CH) ; 139.8 (CH) ; 125.3 (C) ; 123.4 (CH) ; 115.3 (CH) ; 113.1 (CH) ; 112.5 (CH) ; 111.2 (CH) ; 61.1 (CH_2) ; 55.6 (CH_3)

F19. (1R,2S,5R)-2-isopropyl-5-méthylcyclohéxyl (E)-3-(4-hydroxy-3-méthoxyphényl)acrylate [ix]

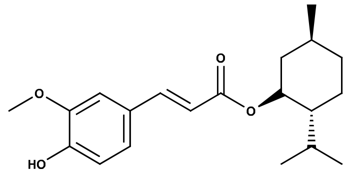

Yield : 12 %
Aspect : pinky oil
^{1}H NMR (400 MHz, CDCl3) δ ppm : 7.60 (d, 1H, CH, J = 15.6 Hz) ; 7.06 (dd, 1H, CH, J = 1.2/8.4 Hz) ; 7.03 (d, 1H, CH, J = 1.3 Hz) ; 6.91 (d, 1H, CH, J = 8.4 Hz) ; 6.28 (d, 1H, CH, J =

16.0 Hz) ; 5.88 (bs, 1H, OH) ; 4.82 (dt, 1H, CH, J=4.4/10.4 Hz) ; 3.92 (s, 3H, CH_3) ; 2.06 (m, 1H, CH) ; 1.93 (m, 1H, CH) ; 1.70 (m, 2H, 2xCH) ; 1.55-1.41 (m, 2H, 2xCH) ; 1.05 (m, 2H, 2xCH) ; 0.91 (dd, 6H, 2xCH_3, J= 2/6.4 Hz) ; 0.89 (m, 1H, CH) ; 0.79 (d, 3H, CH_3, J=7.2 Hz)

ATF 20. 3,7-dimethyloct-6-èn-1-yl (2E)-3-(4-hydroxy-3-methoxyphényl)acrylate

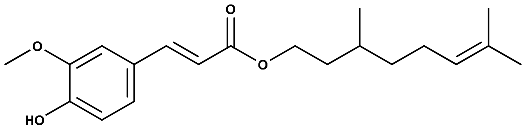

Yield : 57 %

Aspect : colourless oil

TLC Rf : 0.55 (*n*-heptane/ ethyle acetate : 50/50)

^{1}H NMR (400 MHz, $CDCl_3$) δ ppm : 7.60 (d, 1H, CH, J= 15.6 Hz) ; 7.07 (dd, 1H, CH, J=1.2/8.0 Hz) ; 7.03 (d, 1H, CH, J=1.6 Hz) ; 6.91 (d, 1H, CH, J=8.4 Hz) ; 6.29 (d, 1H, CH, J=16.0 Hz) ; 5.88 (s, 1H, OH) ; 5.10 (t, 1H, CH, J=6.8 Hz) ; 4.26-4.20 (m, 2H, CH_2) ; 3.93 (s, 3H, CH_3) ; 2.04-1.96 (m, 2H, CH_2) ; 1.77- 1.49 (m, 3H, CH_2+CH) ; 1.68 (s, 3H, CH_3) ; 1.61 (s, 3H, CH_3) ; 1.41 -1.19 (m, 2H, CH_2) ; 0.95 (d, 3H, CH_3, J=6.4 Hz)

13C NMR (100 MHz, CDCl3) δ ppm : 167.3 (CO) ; 147.9 (CO) ; 146.7 (CO) ; 144.6 (CH) ; 131.3 (C) ; 127.0 (C) ; 124.6 (CH) ; 123.0 (CH) ; 115.6 (CH) ; 114.7 (CH) ; 109.3 (CH) ; 62.9 (CH_2) ; 55.9 (CH_3) ; 36.9 (CH_2) ; 35.5 (CH_2) ; 29.5 (CH) ; 25.7 (CH_3) ; 25.4 (CH_3) ; 19.4 (CH_2) ; 17.6 (CH_3)

Synthesis of F21.

Trans-ferulic acid (5.15 mmol) and *para*-toluene sulfonic acid (0.51 mmol, 0.1 eq) were dissolved in 15 mL of methanol. The reaction mixture was placed under magnetic stirring and reflux. The progress of esterification was monitored by TLC, and after complete conversion of *trans*-ferulic acid, the solvent was evaporated. The crude reaction mixture was then solubilized in 30 mL of ethyl acetate and washed with a saturated $NaHCO_3$ solution (3x15 mL) followed by a saturated NaCl solution (3x15 mL). The organic phase was dried using anhydrous magnesium sulfate, filtered, and concentrated under vacuum. Without prior purification, methyl ferulate (4.80 mmol) and triethylamine (9.6 mmol, 2 eq) were solubilized with 10 mL of THF in a two-neck flask. A mixture of 10 mL of THF and acetyl chloride (4.80 mmol, 1 eq) was then added dropwise to the reaction medium at 0-5°C. The system was then maintained at room temperature until complete conversion of the methyl ferulate. The triethylammonium salt was

then removed by filtration and the solvent was evaporated. The solid thus collected was washed with ether to obtain the pure product F21.

F21. Méthyl (E)-3-(3,4-diméthoxyphényl)acrylate [x]

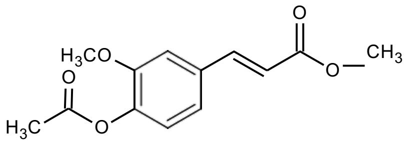

Yield : 78 %

Aspect : white solid

Melting point: 122°C

^{1}H NMR (400 MHz, $CDCl_3$) δ ppm : 7.65 (d, 1H, CH, J = 16.0 Hz) ; 7.13 (d, 1H, CH, J = 1.9 Hz) ; 7.11 (dd, 1H, CH, J = 2.0/4.7 Hz) ; 7.05 (d, 1H, CH, J = 8.2 Hz) ; 6.38 (d, 1H, CH, J = 16.0 Hz) ; 3.86 (s, 3H, CH_3) ; 3.81 (s, 3H, CH_3) ; 2.32 (s, 3H, CH_3)

^{13}C NMR (100 MHz, $CDCl_3$) δ ppm : 168.8 ; 167.2 ; 151.4 ; 144.1 ; 141.4 ; 133.3 ; 123.2 ; 121.2 ; 118.0 ; 111.2 ; 55.9 ; 51.7 ; 20.6

ATF19. Pentyl (2E)-3-(4-hydroxy-3,5-dimethoxyphenyl)acrylate

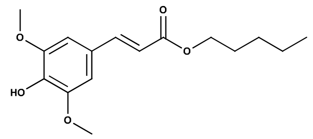

Yield : 13 %

Aspect : yellow oil

^{1}H NMR (400 MHz, DMSO) δ ppm : 8.94 (bs, 1H, OH) ; 7.54 (d,1H, CH, J=16.1 Hz) ; 7.03 (s, 2H, 2xCH) ; 6.53 (d, 1H, CH, J=16.1 Hz) ; 3.80 (s, 6H, 2xCH_3) ; 4.12 (t, 2H, CH_2, J=7.0 Hz) ; 1.63 (quint., 2H, CH_2) ; 1.38-1.27 (m, 4H, 2xCH_2) ; 0.89 (t, 3H, CH_3, J=7.3 Hz)

III. ^{1}H and ^{13}C NMR spectra of the compounds

F1. (2*E*)-3-[4-(acetyloxy)-3-methoxyphenyl]prop-2-enoic acid

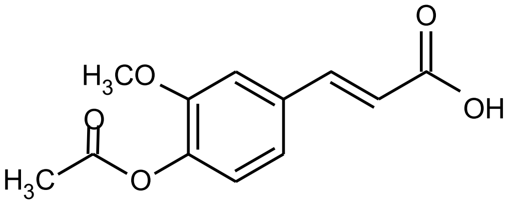

^{1}H NMR (400 MHz, DMSO)

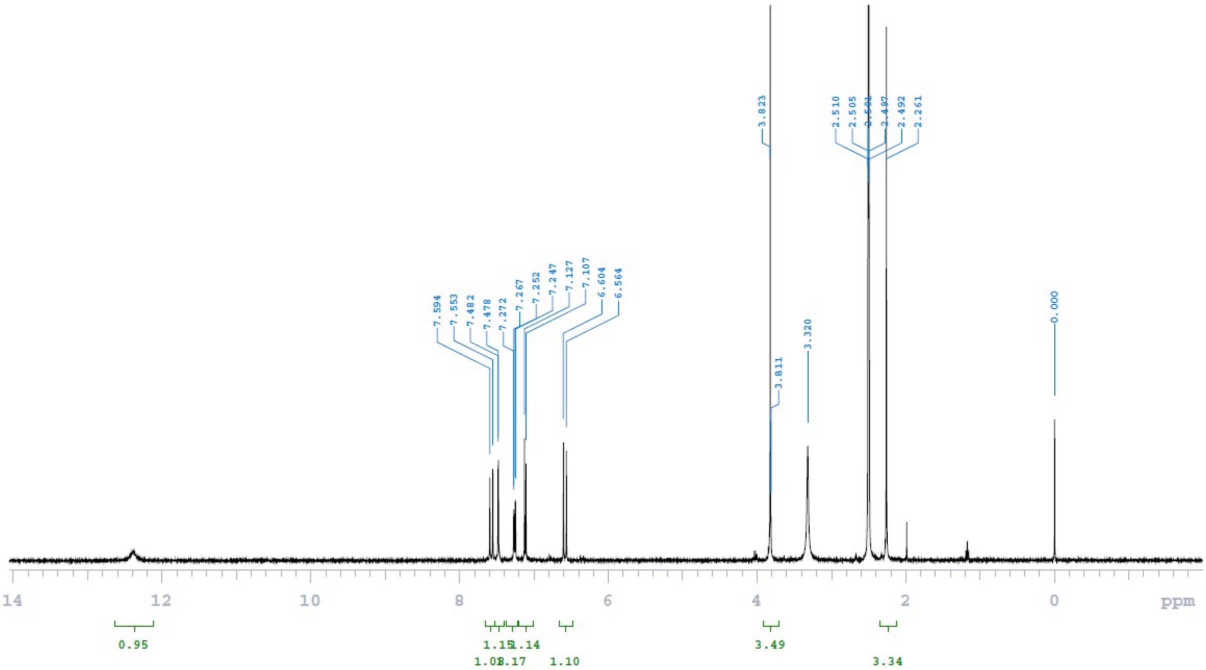

^{13}C NMR (100 MHz, DMSO)

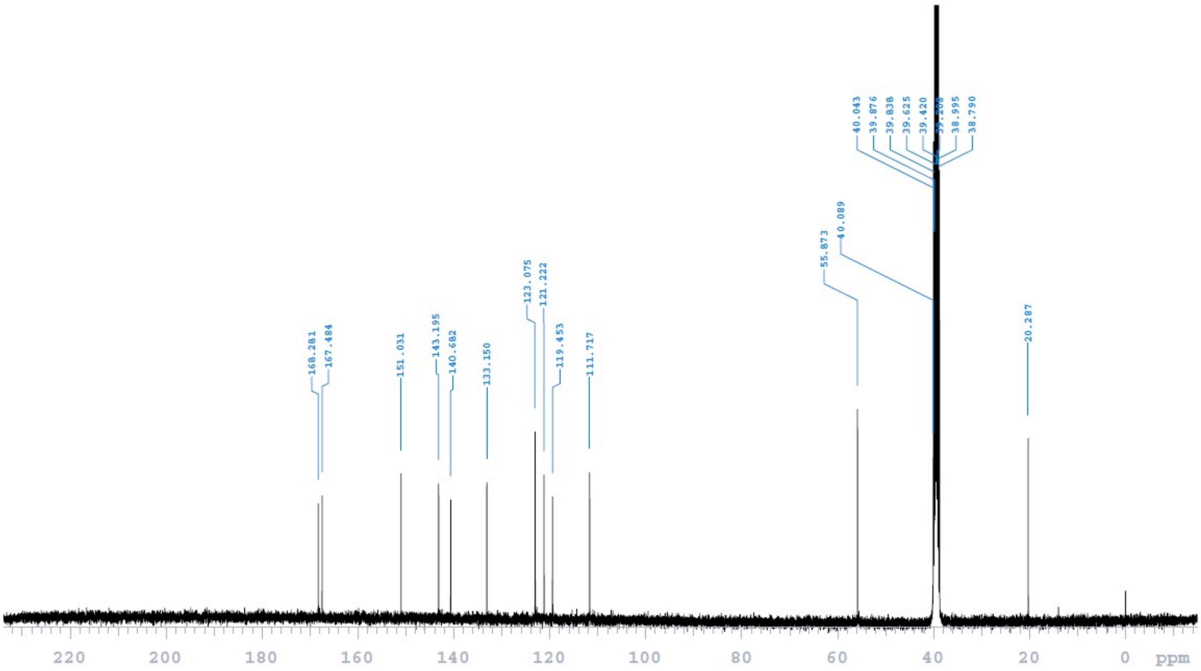

F2. (2*E*)-3-[3-methoxy-4-(propanoyloxy)phenyl]prop-2-enoic acid

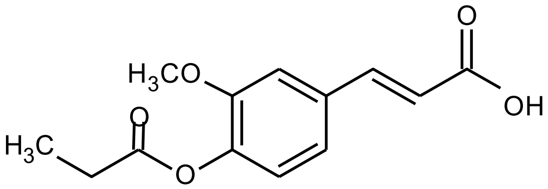

^{1}H NMR (400 MHz, DMSO)

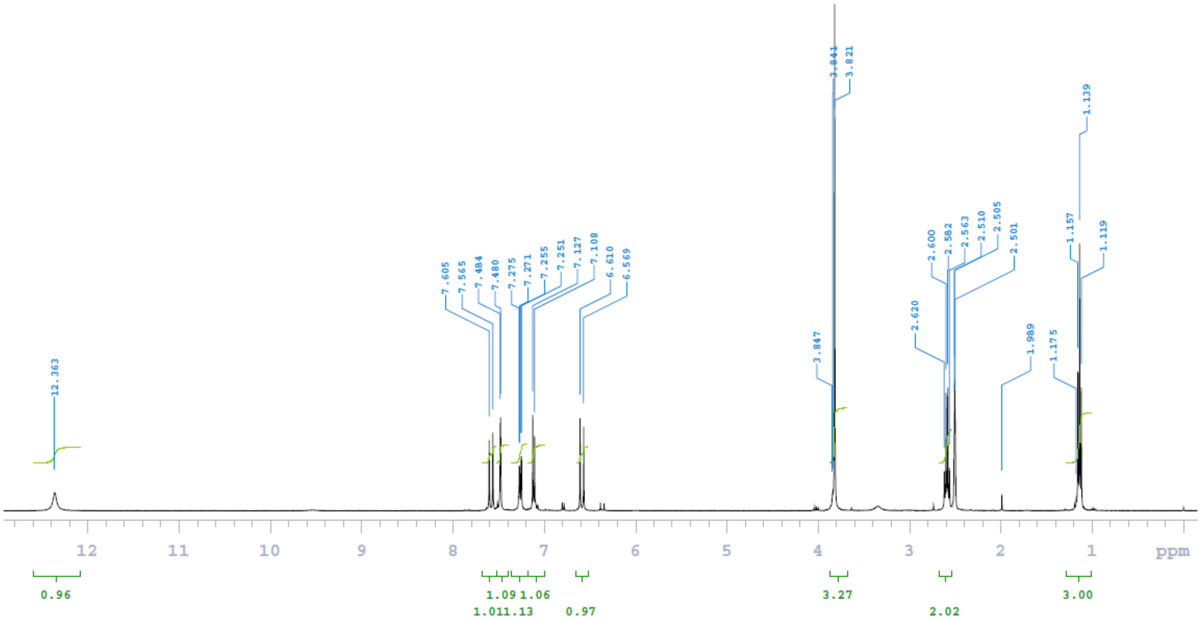

^{13}C NMR (100 MHz, DMSO)

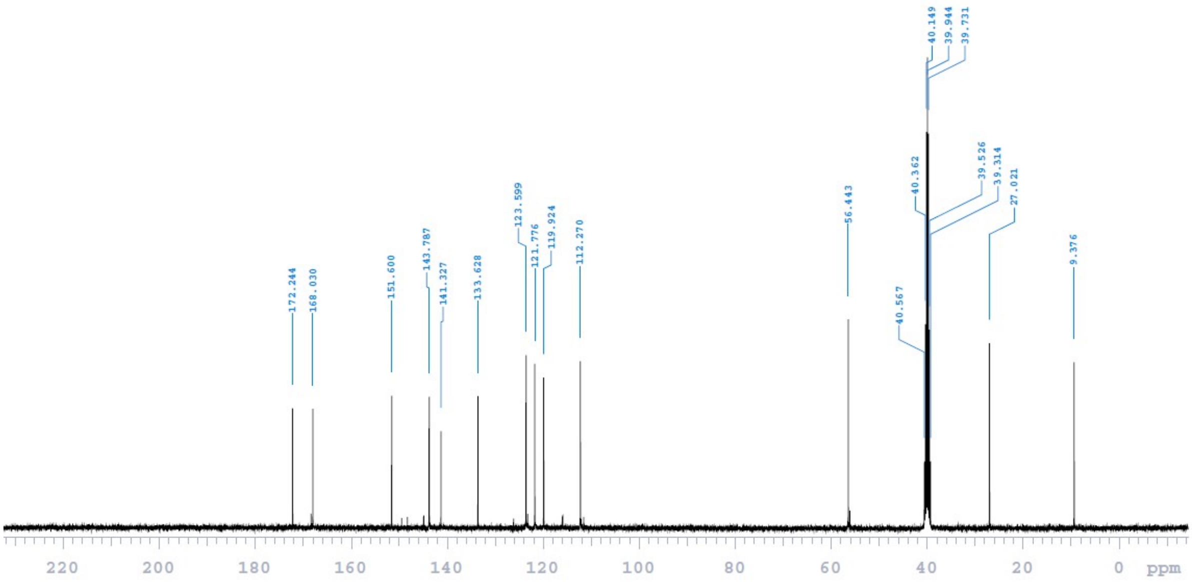

MB22. (2*E*)-3-[4-(butanoyloxy)-3-methoxyphenyl]prop-2-enoic acid

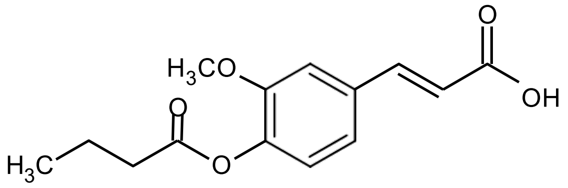

^{1}H NMR (400 MHz, DMSO)

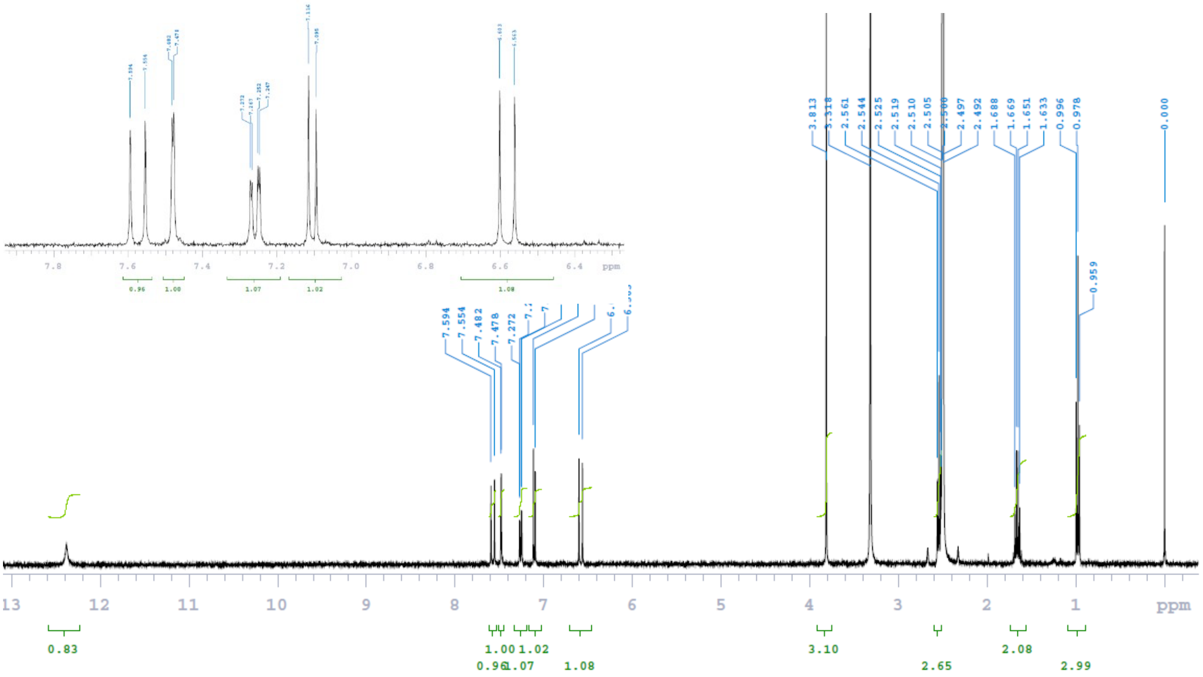

^{13}C NMR (100 MHz, DMSO)

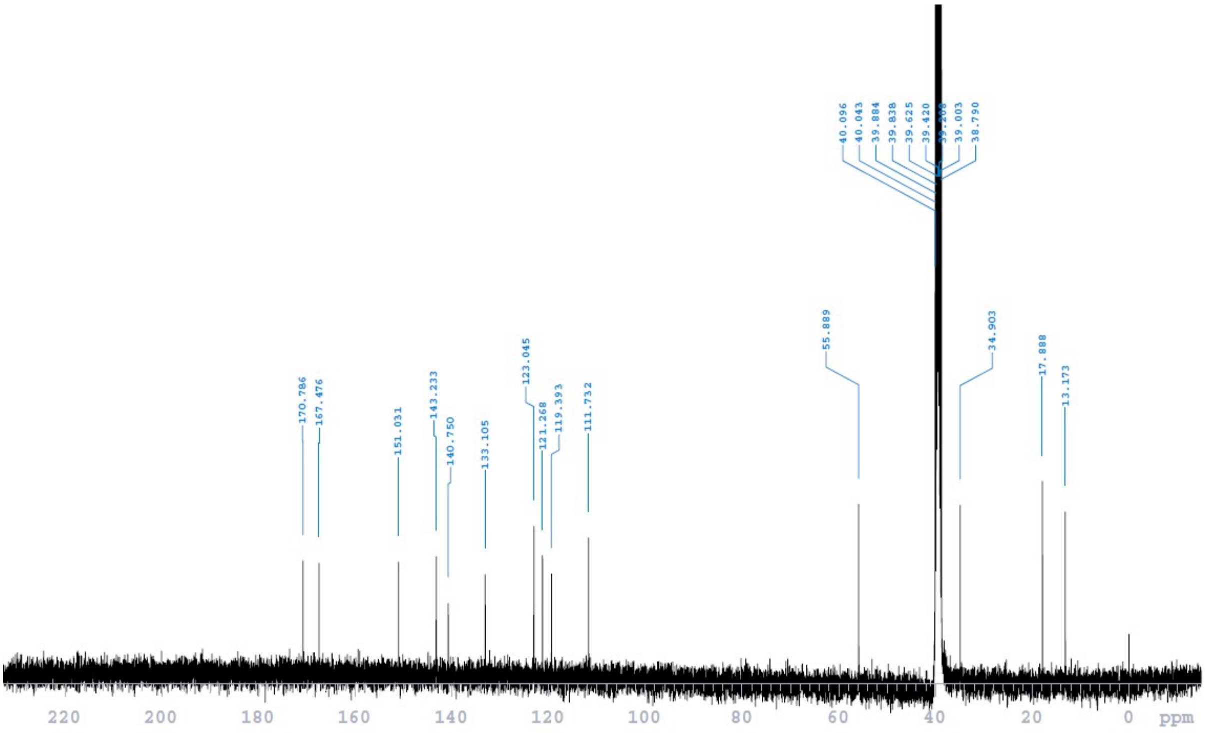

F4. (2*E*)-3-[3-methoxy-4-(pentanoyloxy)phenyl]prop-2-enoic acid

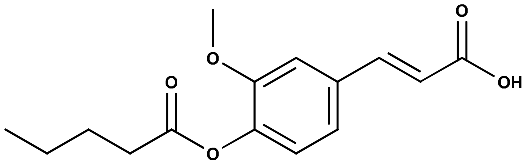

^{1}H NMR (400 MHz, DMSO)

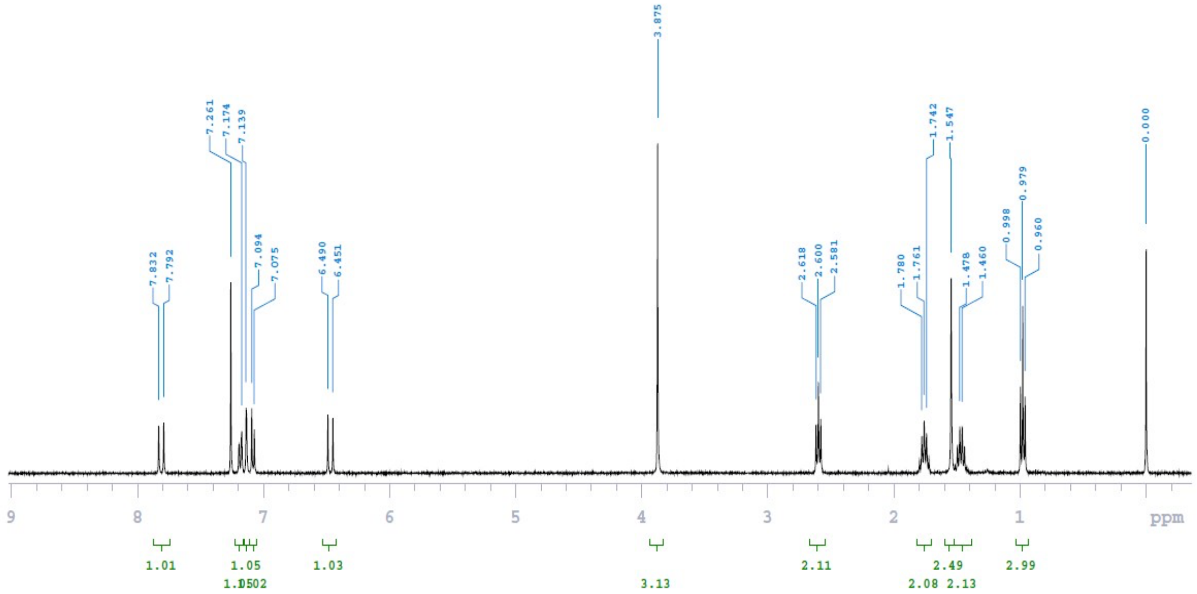

^{13}C NMR (100 MHz, DMSO)

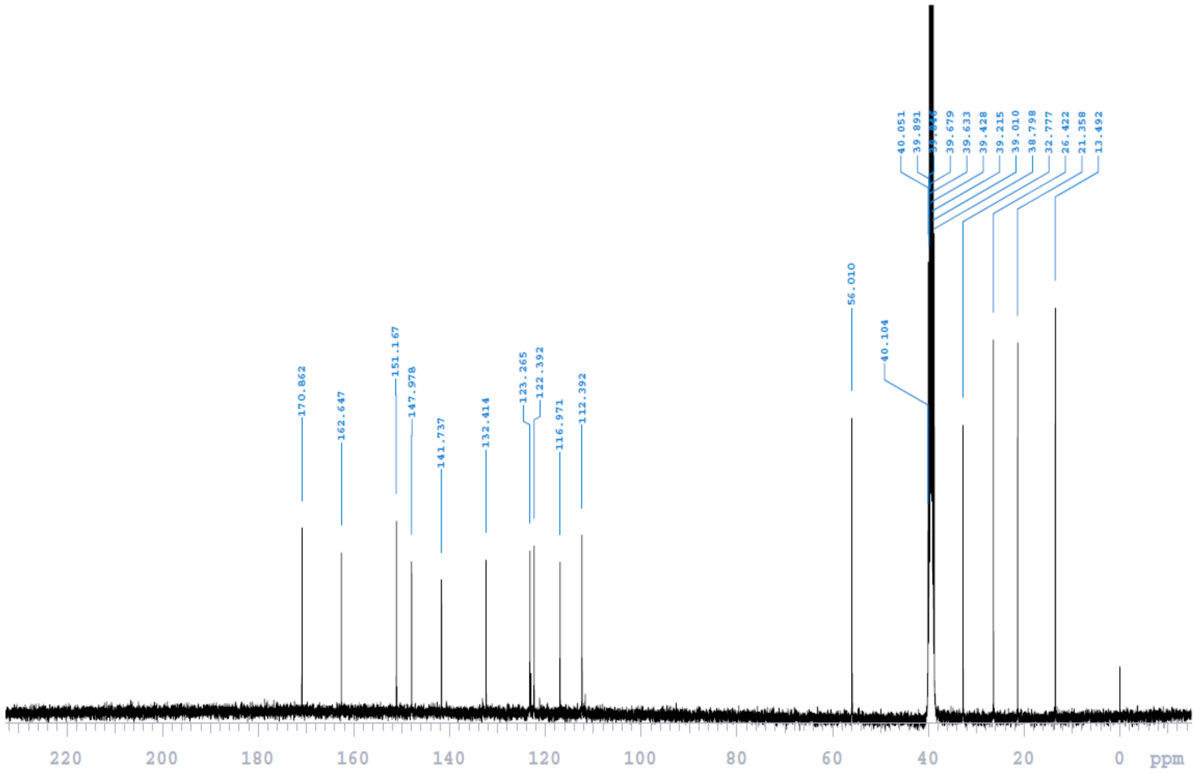

F5. (2*E*)-3-[4-(hexanoyloxy)-3-methoxyphenyl]prop-2-enoic acid

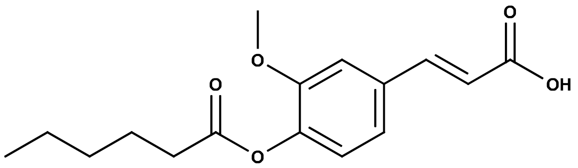

^{1}H NMR (400 MHz, DMSO)

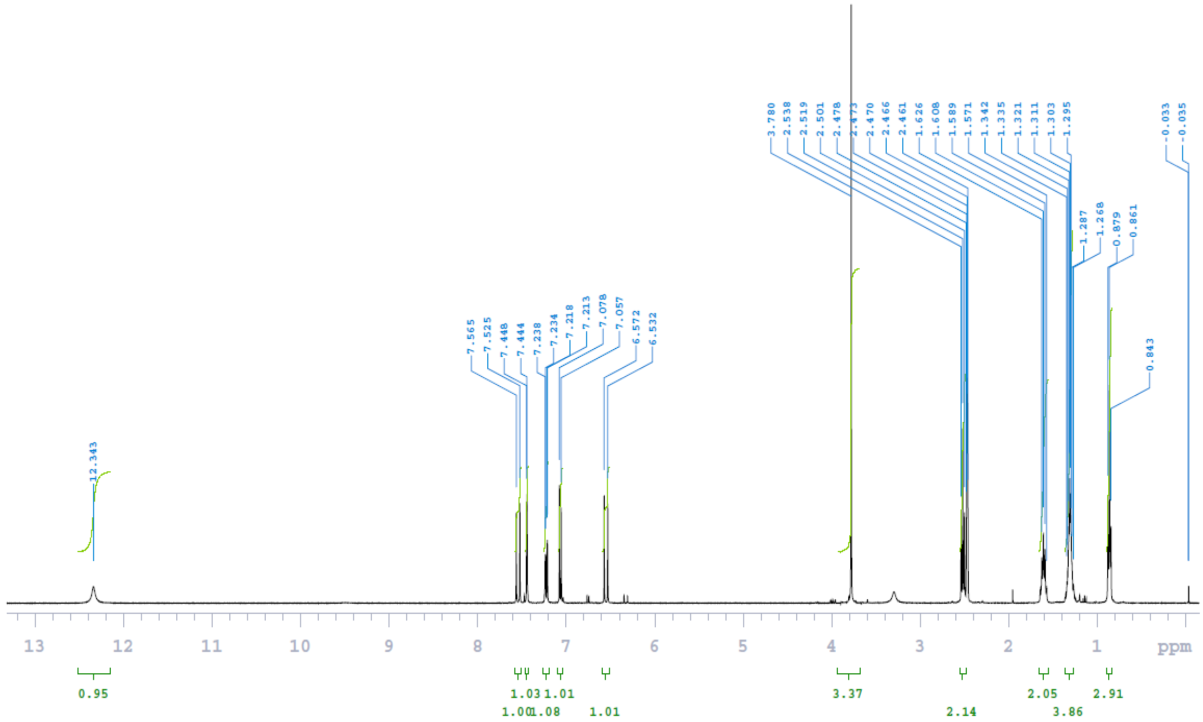

^{13}C NMR (100 MHz, DMSO)

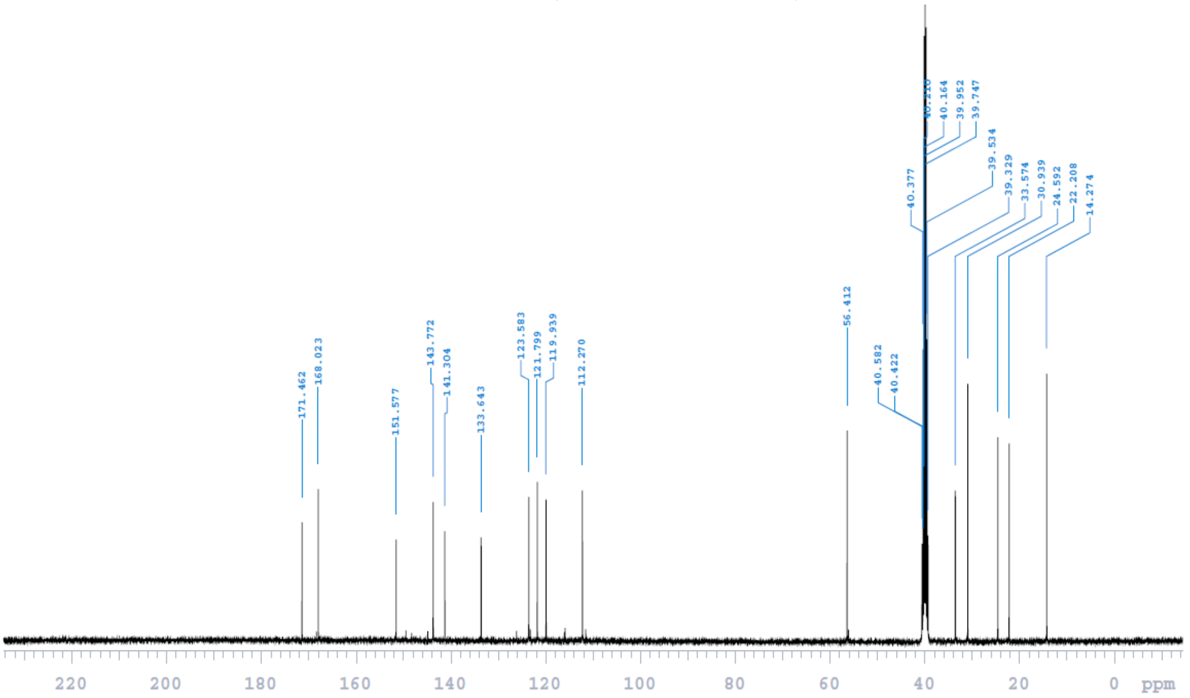

F6. (2*E*)-3-[4-(heptanoyloxy)-3-methoxyphenyl]prop-2-enoic acid

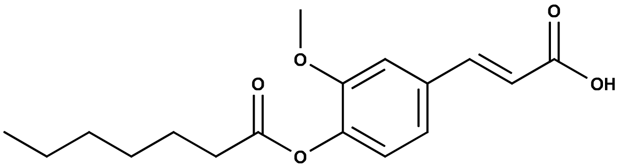

^{1}H NMR (400 MHz, DMSO)

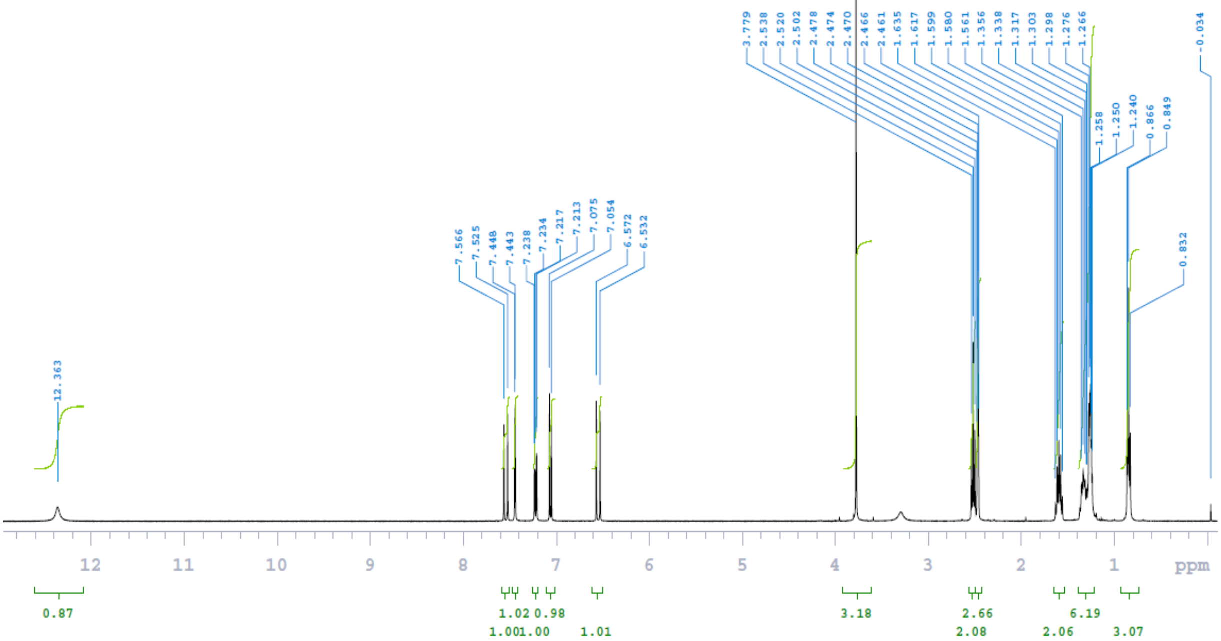

^{13}C NMR (100 MHz, DMSO)

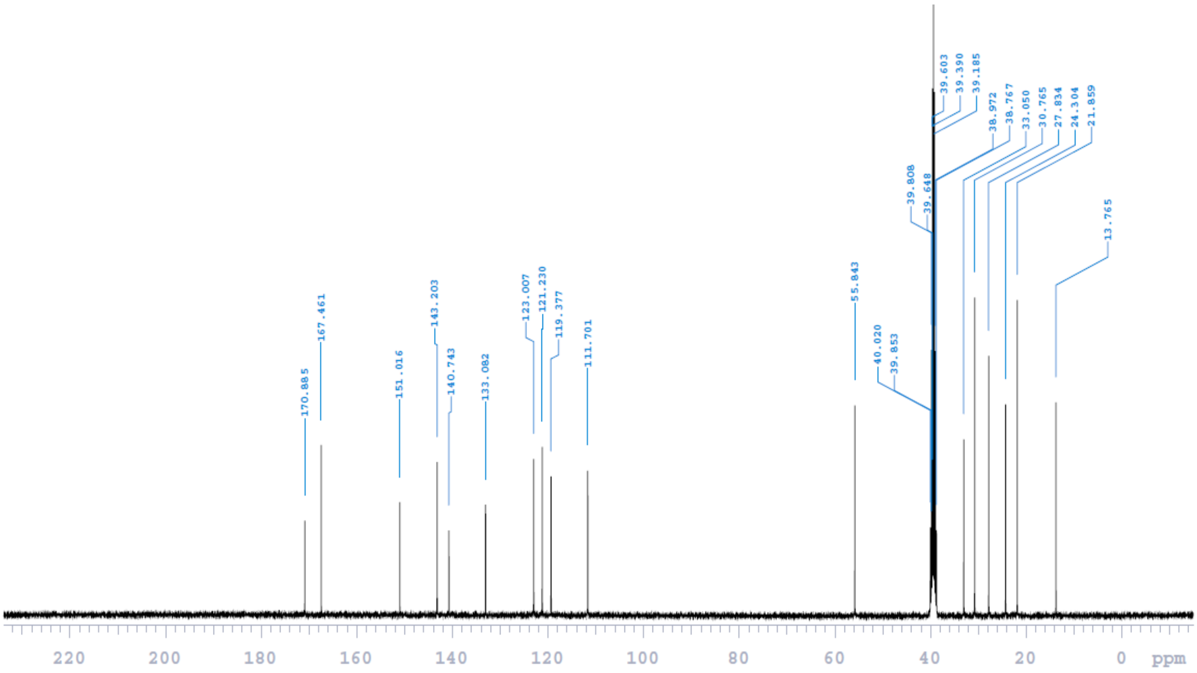

F7. (2*E*)-3-[3-methoxy-4-(octanoyloxy)phenyl]prop-2-enoic acid

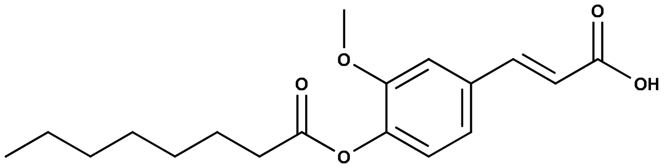

^{1}H NMR (400 MHz, DMSO)

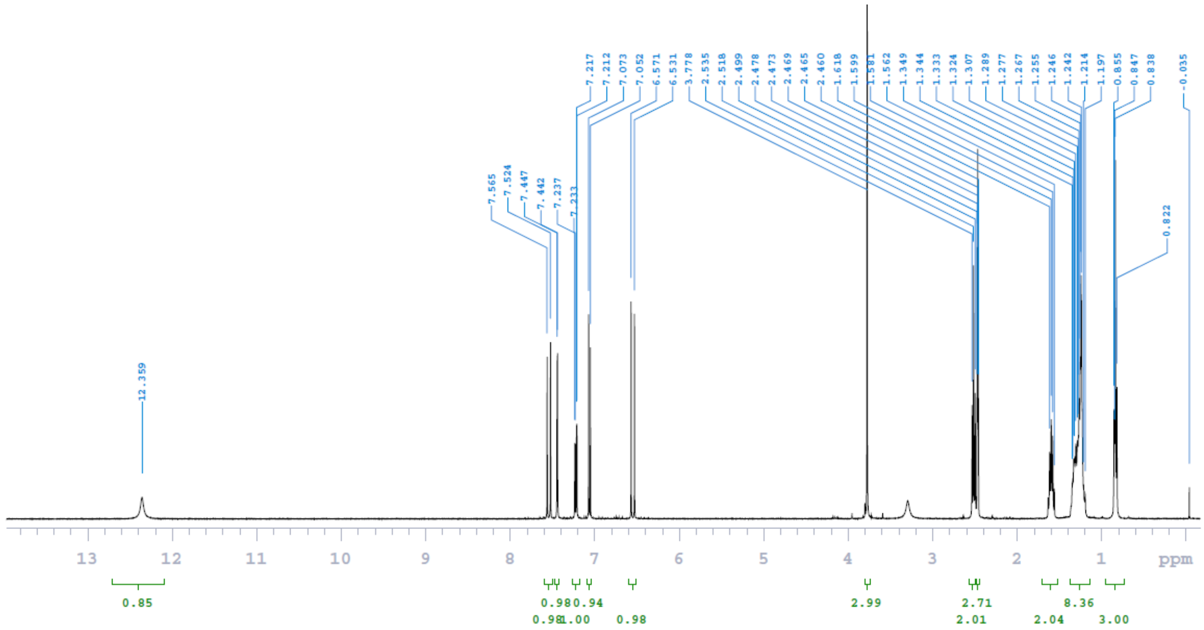

^{13}C NMR (100 MHz, DMSO)

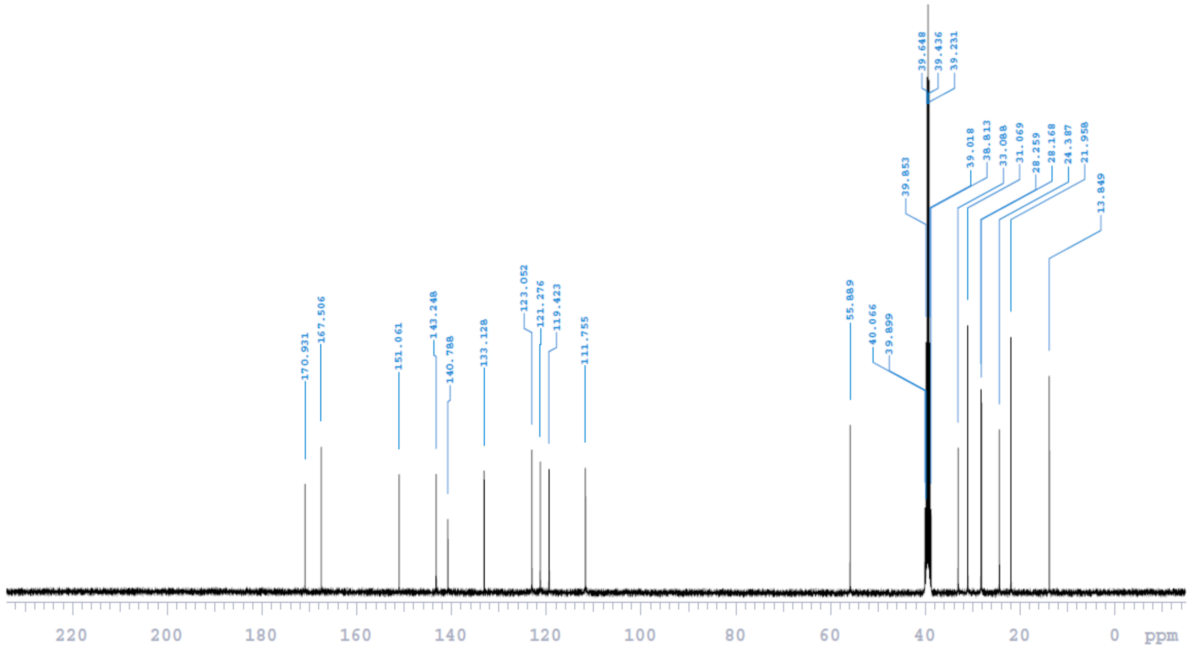

F8. (2*E*)-3-[3-methoxy-4-(nonanoyloxy)phenyl]prop-2-enoic acid

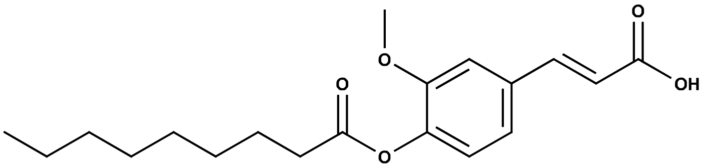

^{1}H NMR (400 MHz, DMSO)

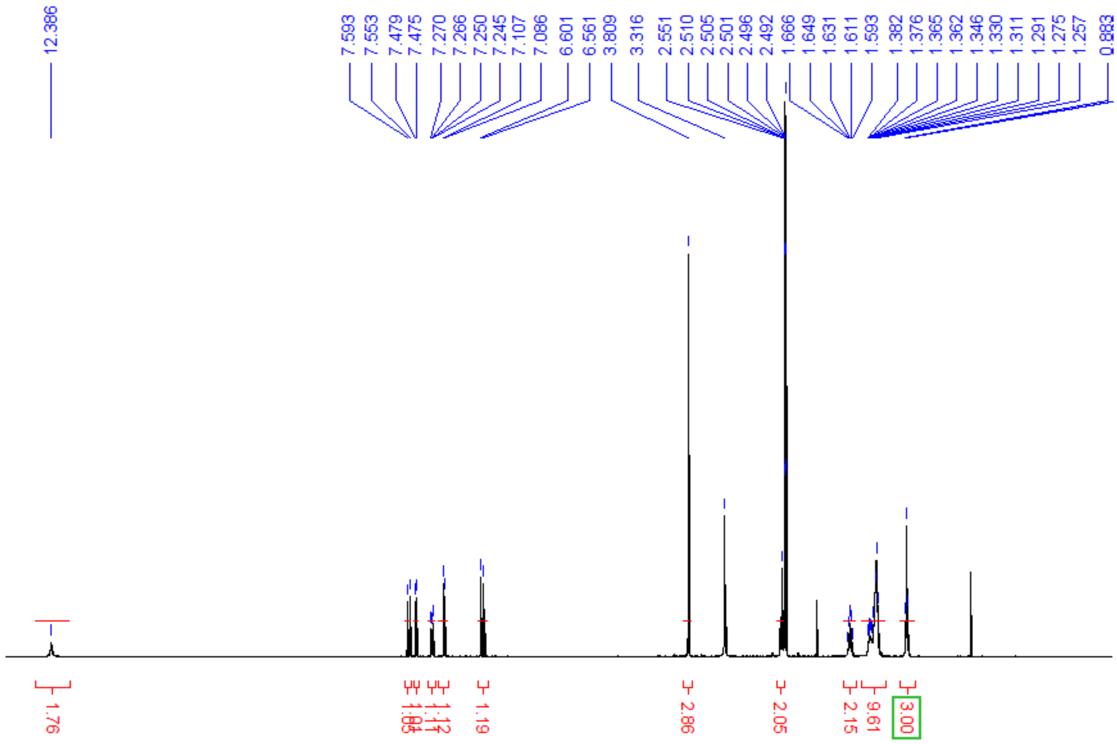

F9. Methyl (2*E*)-3-(4-hydroxy-3-methoxyphenyl)prop-2-enoate

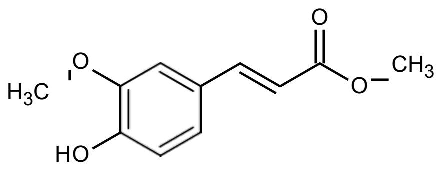

^{1}H NMR (400 MHz, CDCl$_3$)

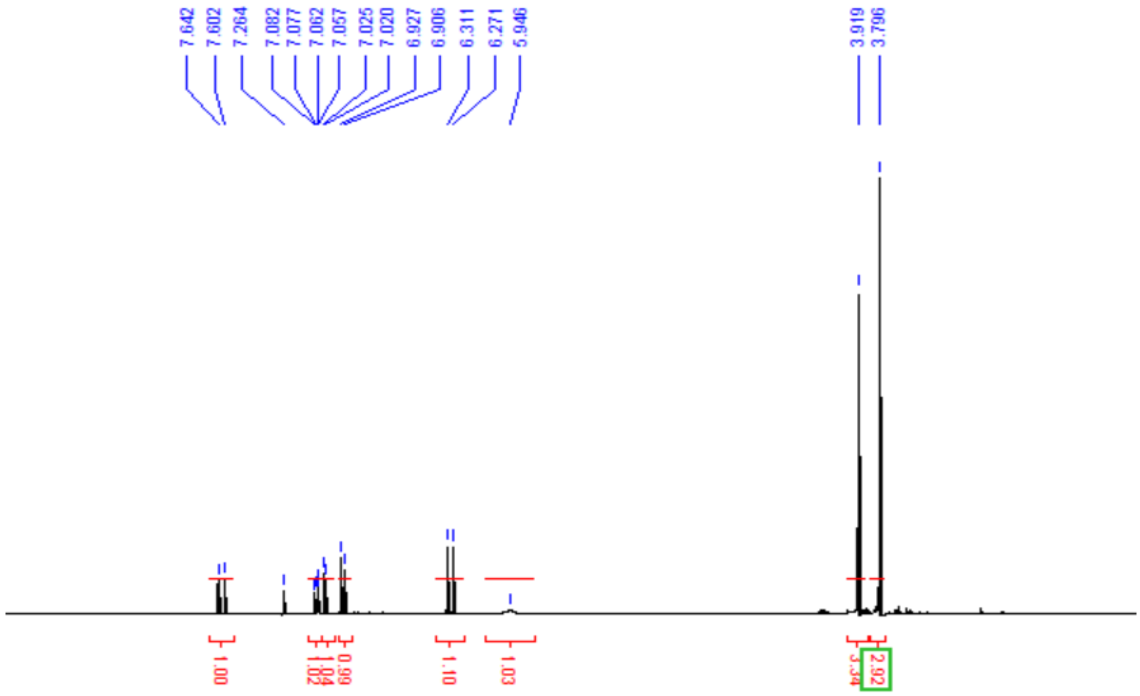

^{13}C NMR (100 MHz, CDCl$_3$)

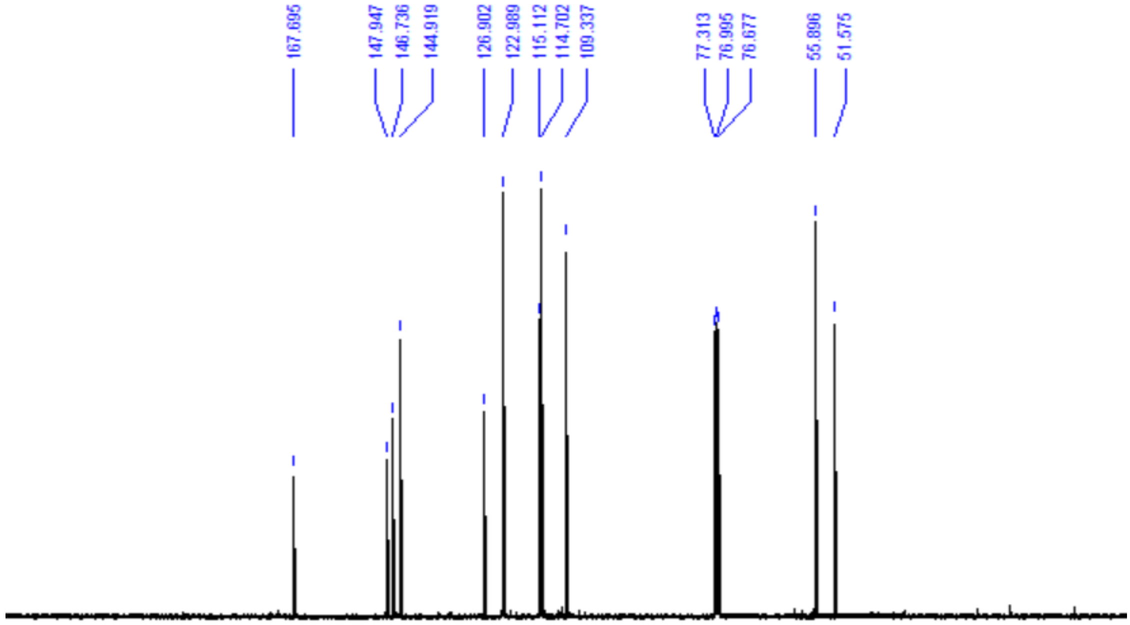

F10. Ethyl(2*E*)-3-(4-hydroxy-3-methoxyphenyl)prop-2-enoate

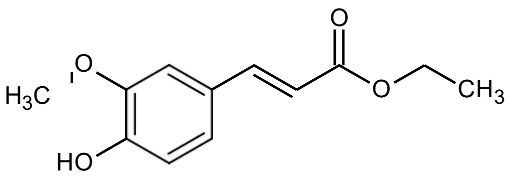

^{1}H NMR (400 MHz, CDCl₃)

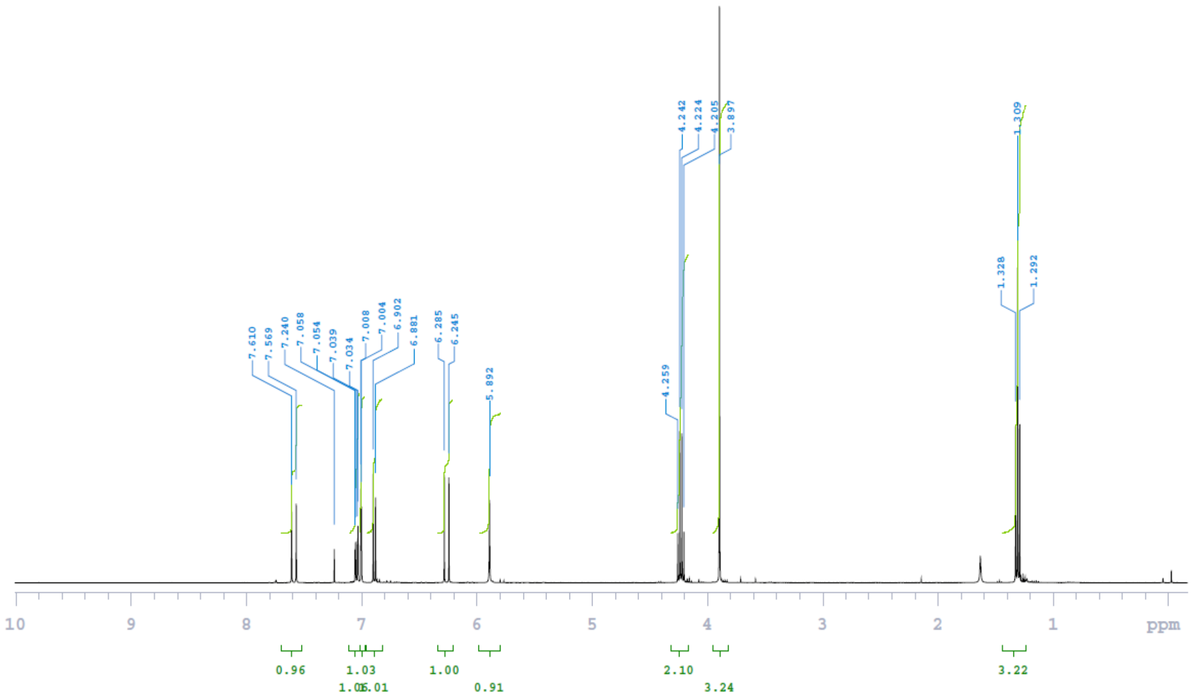

^{13}C NMR (100 MHz, CDCl₃)

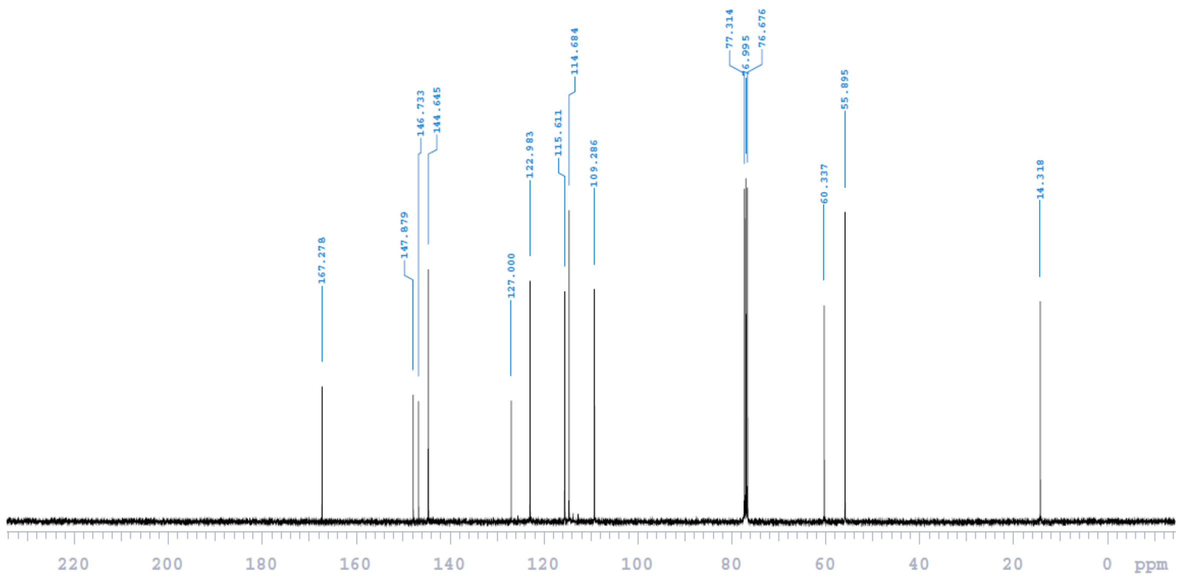

F11. Propyl (2*E*)-3-(4-hydroxy-3-methoxyphenyl)prop-2-enoate)

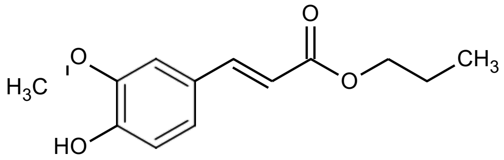

^{1}H NMR (400 MHz, $CDCl_3$)

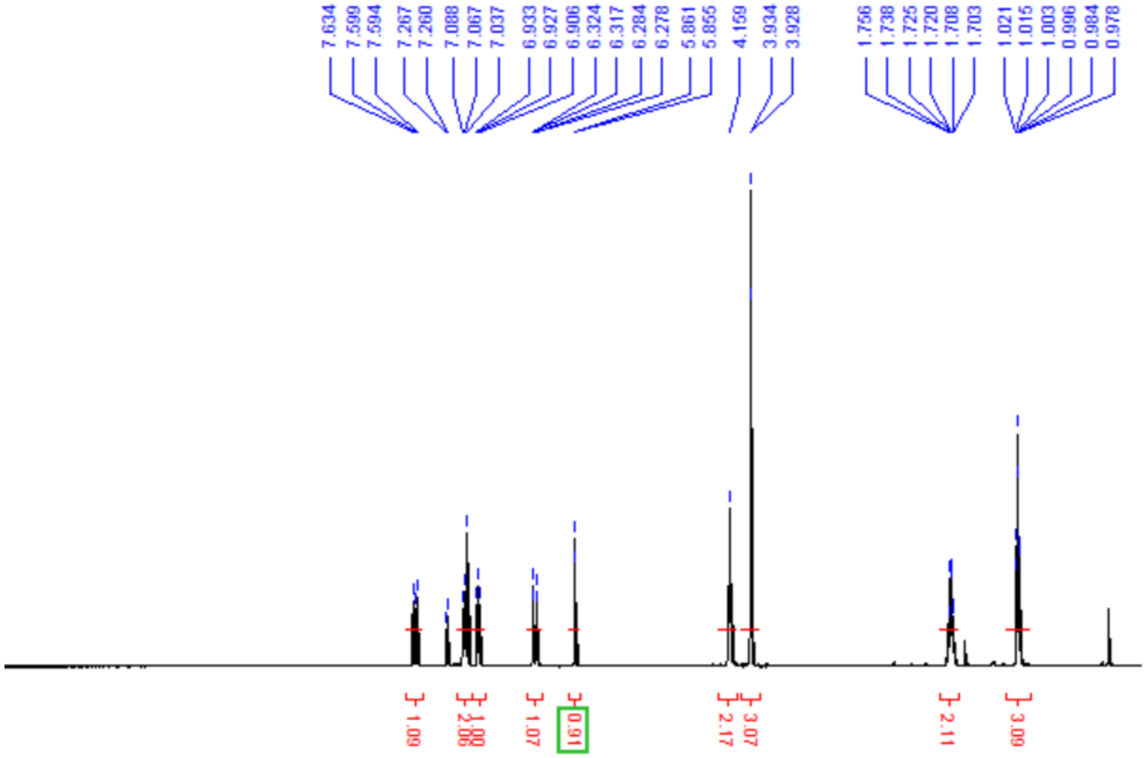

^{13}C NMR (100 MHz, $CDCl_3$)

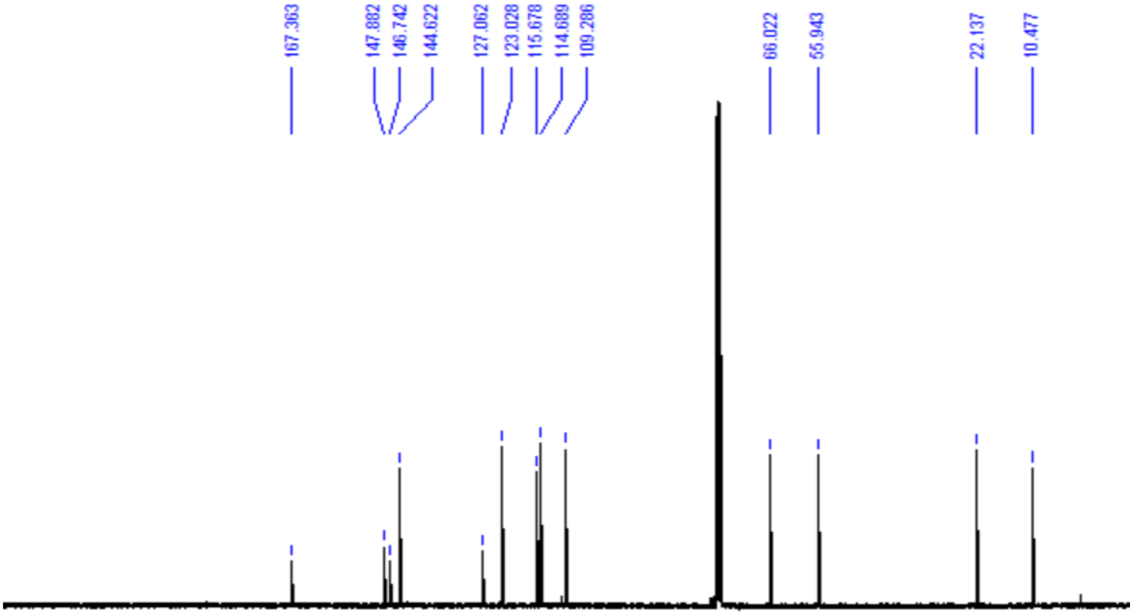

F12. Butyl(2*E*)-3-(4-hydroxy-3-methoxyphenyl)prop-2-enoate

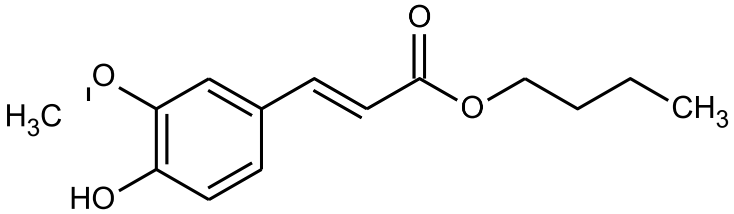

¹H NMR (400 MHz CDCl₃)

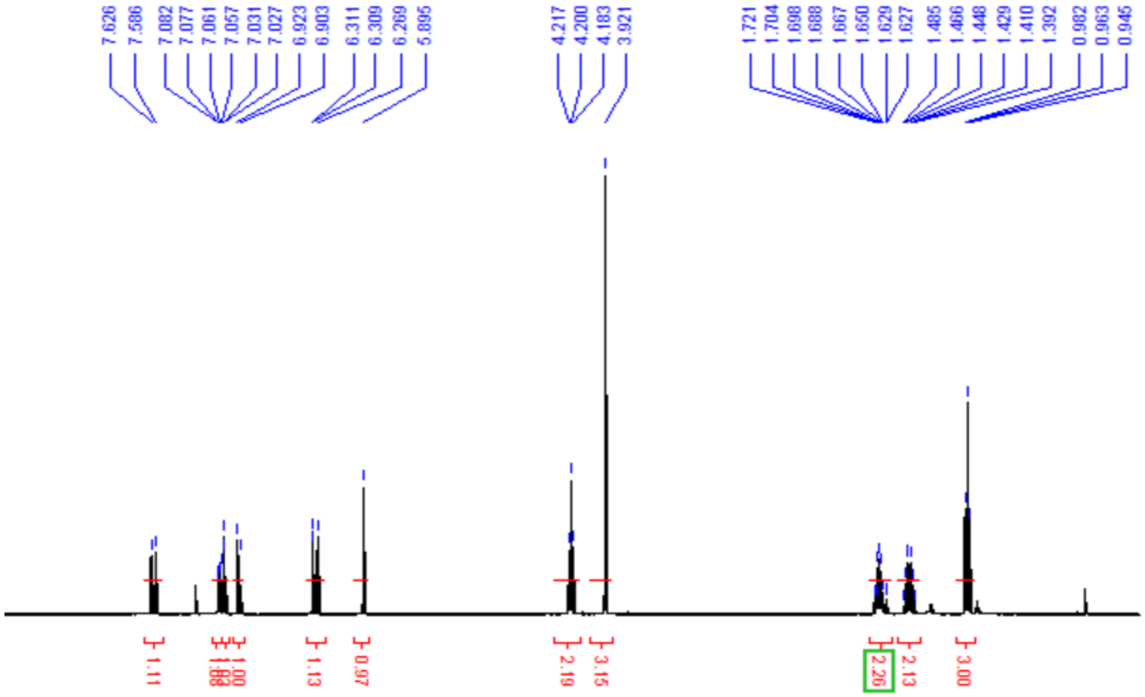

¹³C NMR (100 MHz, CDCl₃)

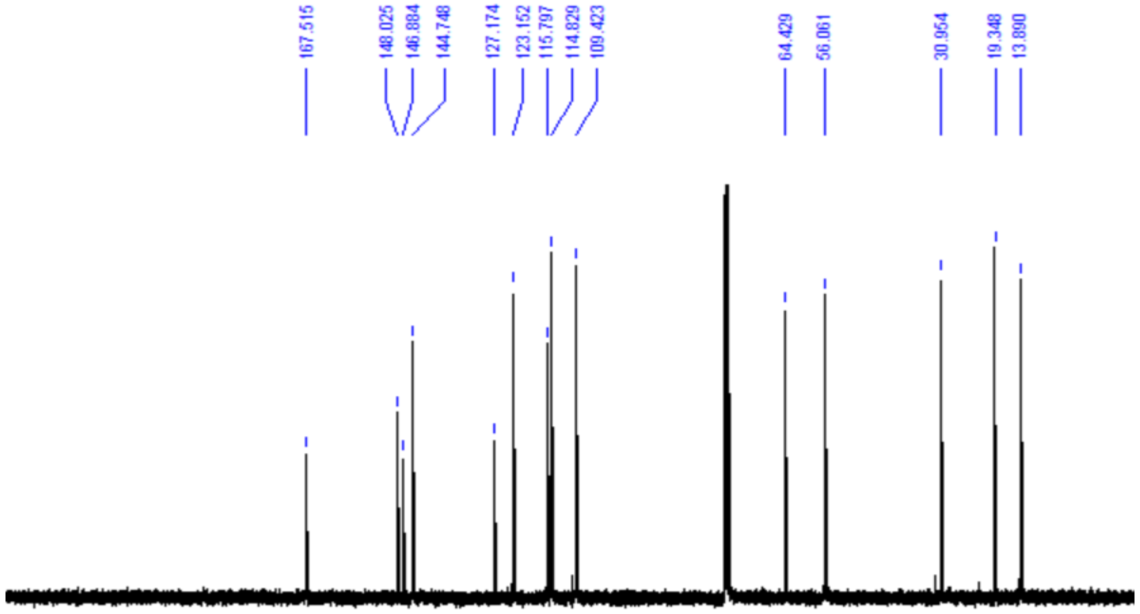

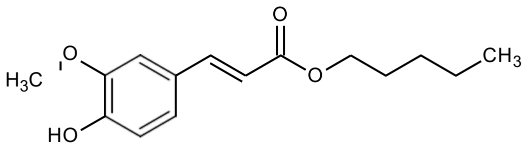

^{1}H NMR (400 MHz, $CDCl_3$)

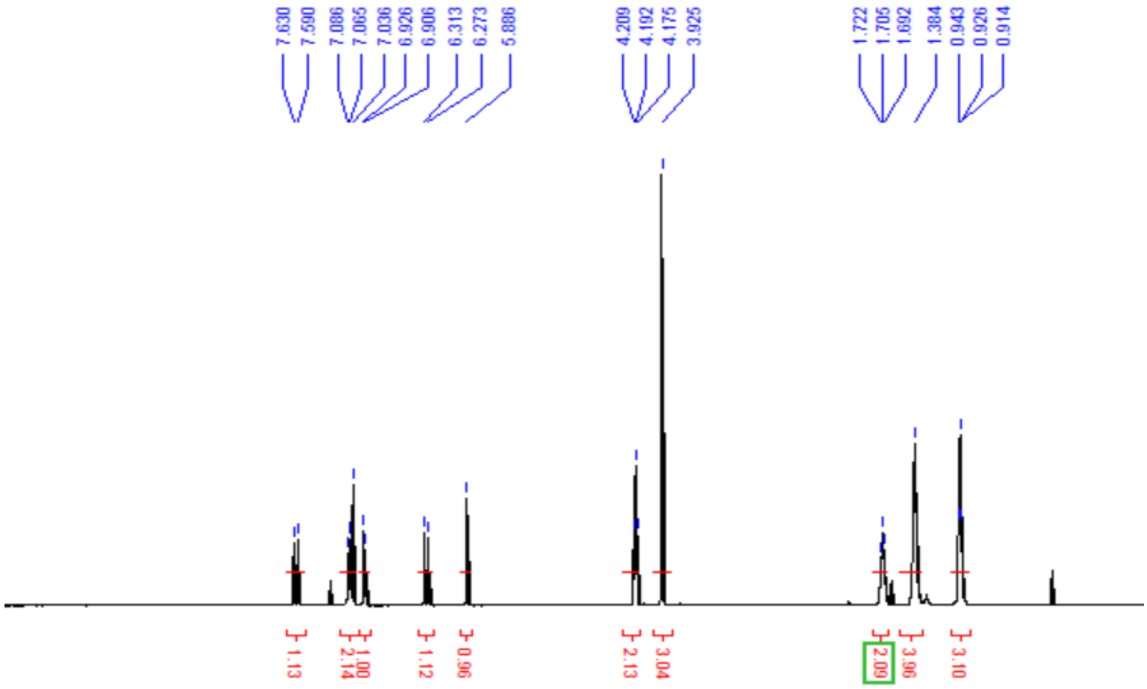

^{13}C NMR (100 MHz, $CDCl_3$)

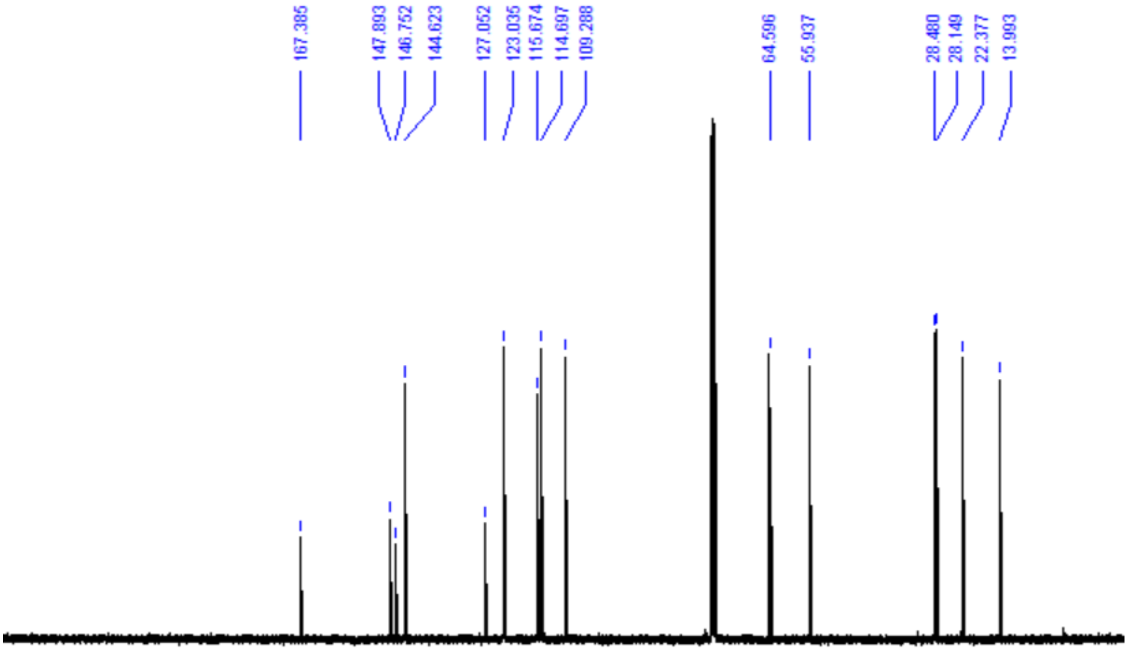

F15. Heptyl (2E)-3-(4-hydroxy-3-méthoxyphényl)prop-2-enoate

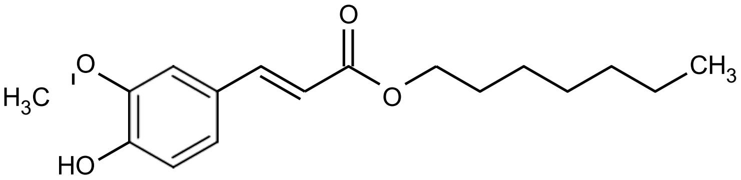

^{1}H NMR (400 MHz, CDCl$_3$)

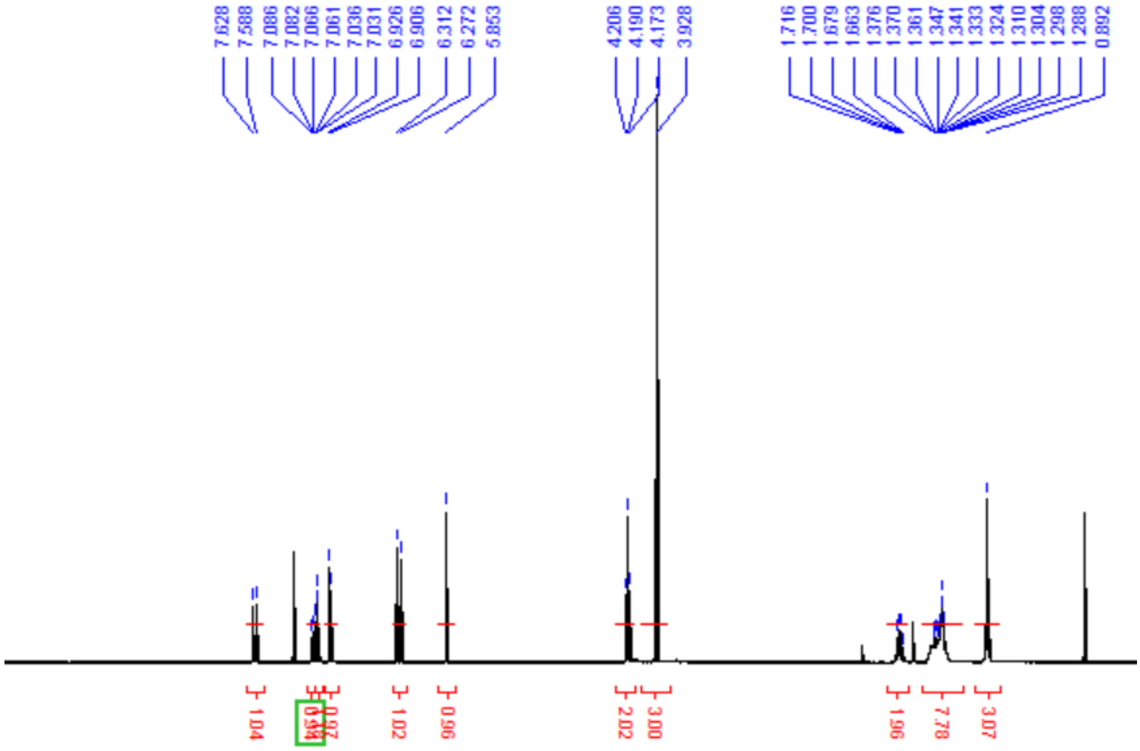

^{13}C NMR (100 MHz, CDCl$_3$)

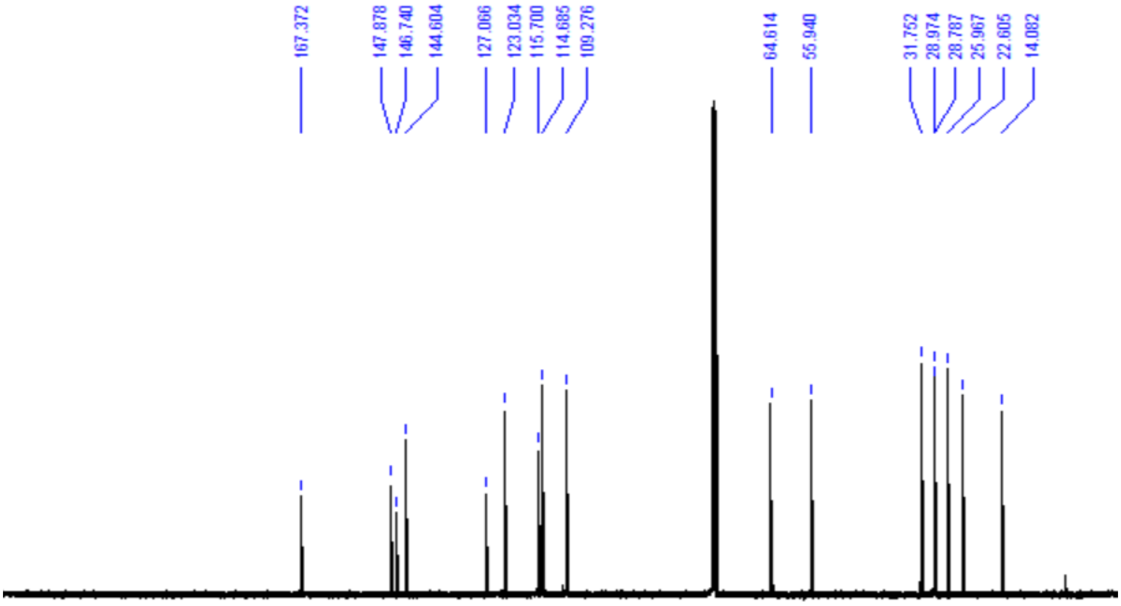

F16. Octyl (2E)-3-(4-hydroxy-3-méthoxyphényl)prop-2-enoate

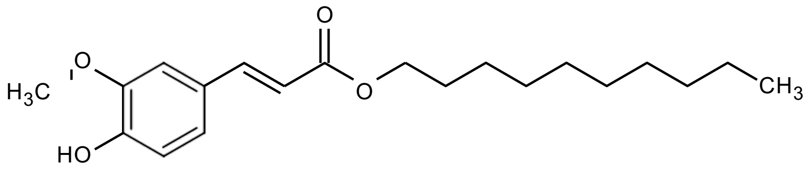

^{1}H NMR (400 MHz, $CDCl_3$)

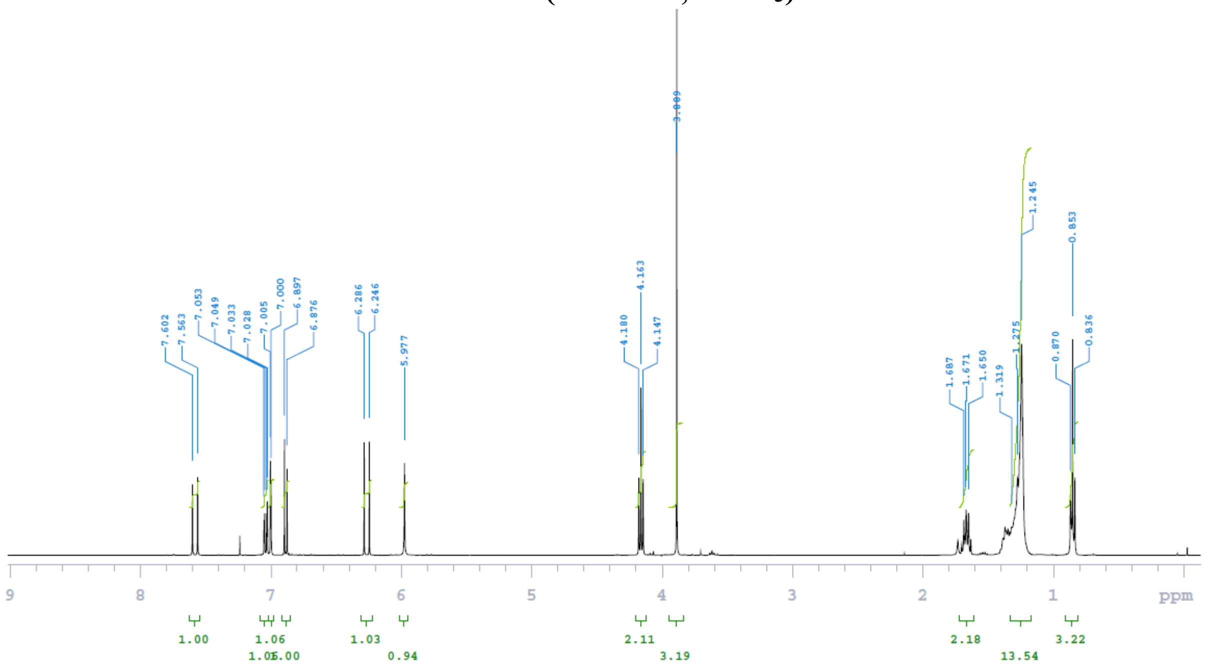

^{13}C NMR (100 MHz, $CDCl_3$)

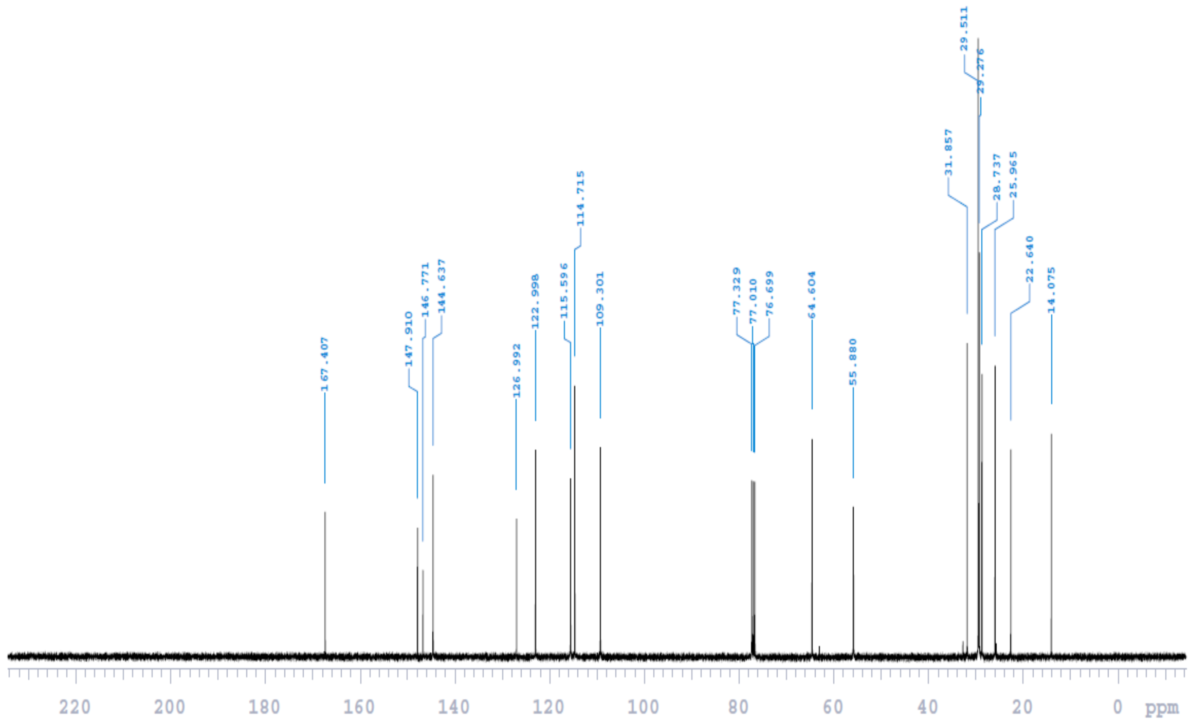

F17. Isopentyl (E)-3-(4-hydroxy-3-méthoxyphényl)prop-2-enoate

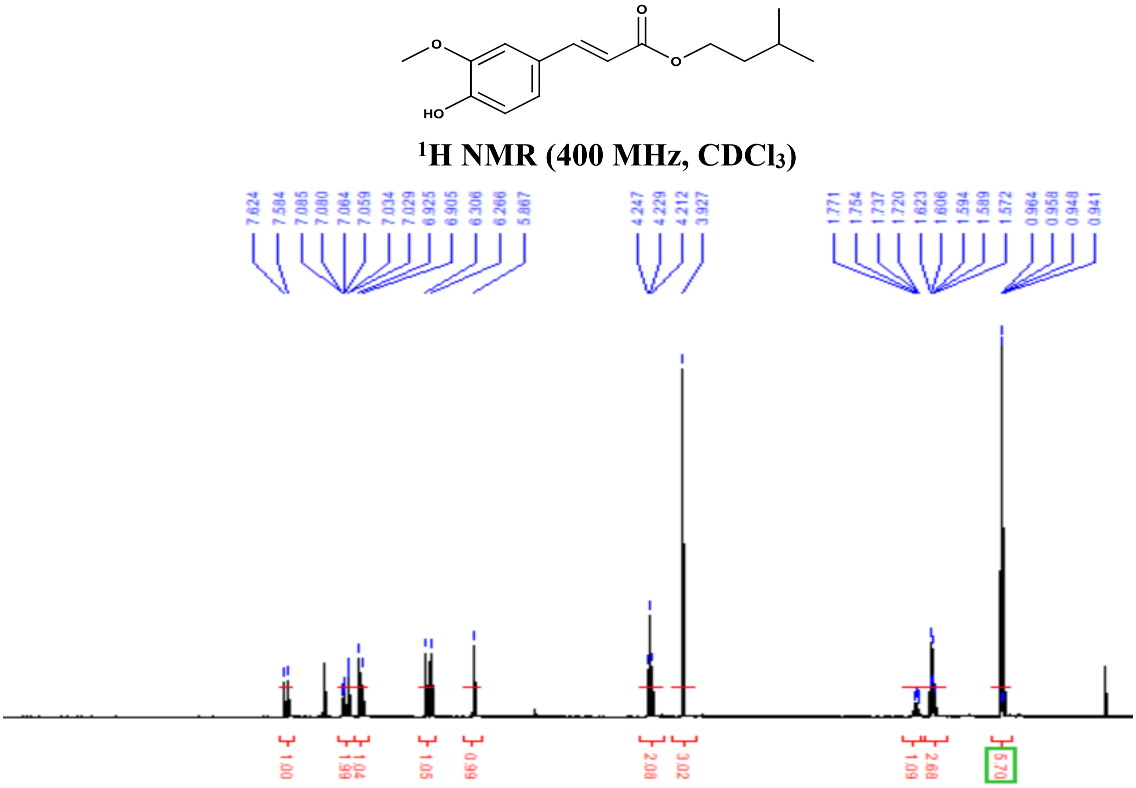

F14. Hexyl (2*E*)-3-(4-hydroxy-3-methoxyphenyl)prop-2-enoate

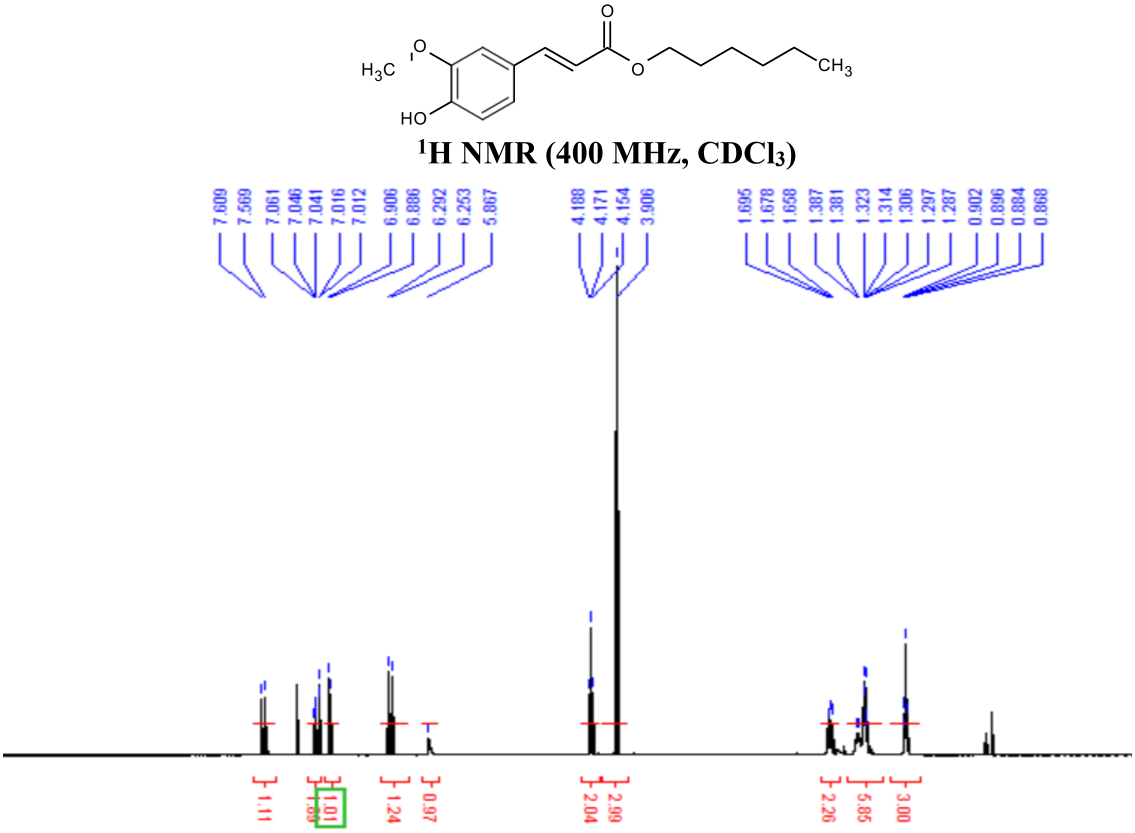

F18. (5-hydroxy-4-oxo-4H-pyran-2-yl)methyl(E)-3-(4-hydroxy-3-methoxyphenyl) acrylate

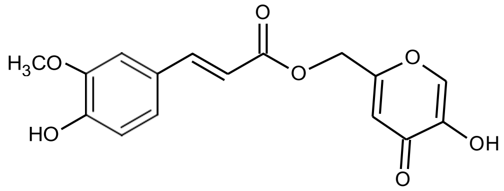

^{1}H NMR (400 MHz, DMSO)

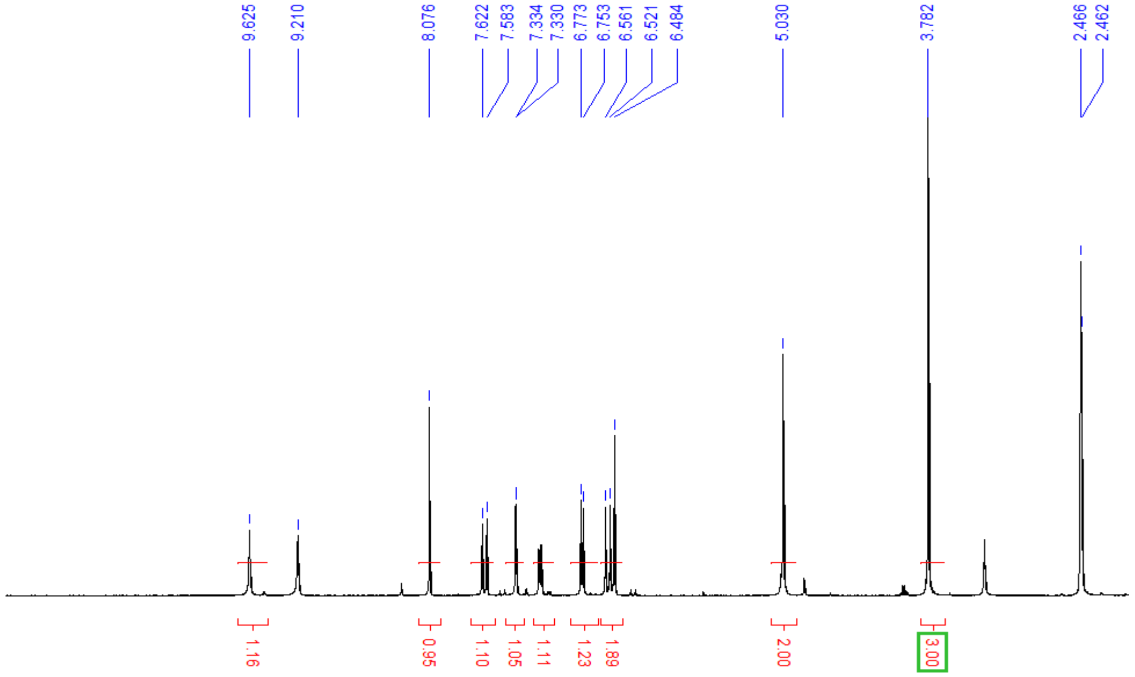

^{13}C NMR (100 MHz, DMSO)

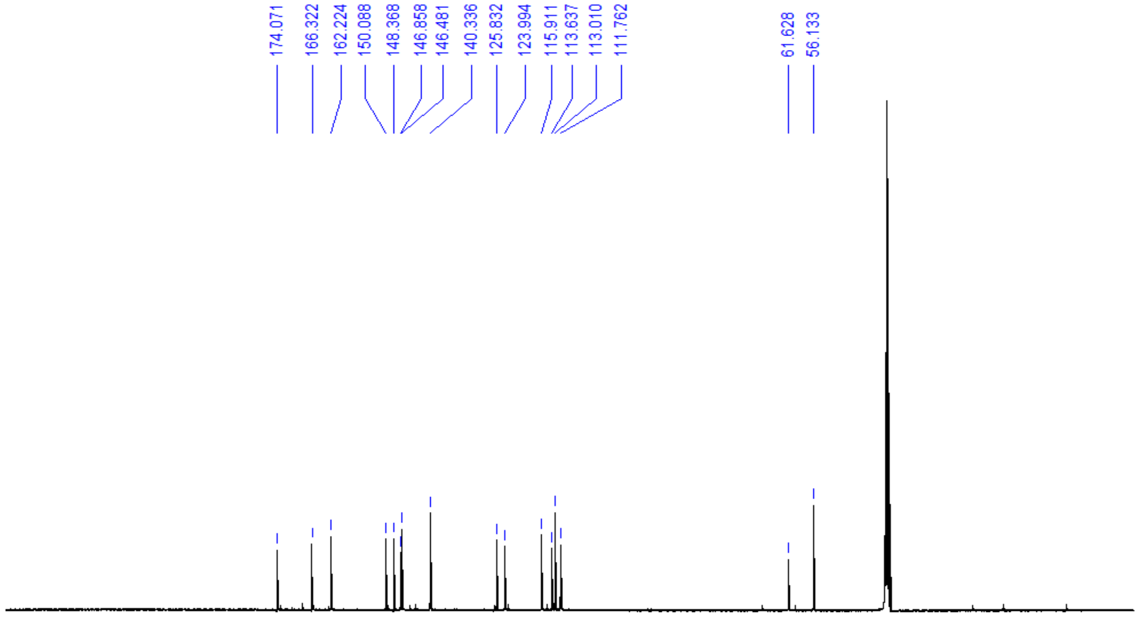

ATF 20. 3,7-dimethyloct-6-èn-1-yl (2E)-3-(4-hydroxy-3-methoxyphényl)acrylate

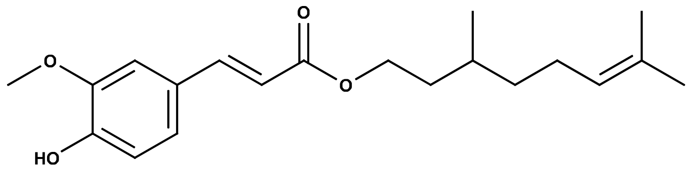

^{1}H NMR (400 MHz, $CDCl_3$)

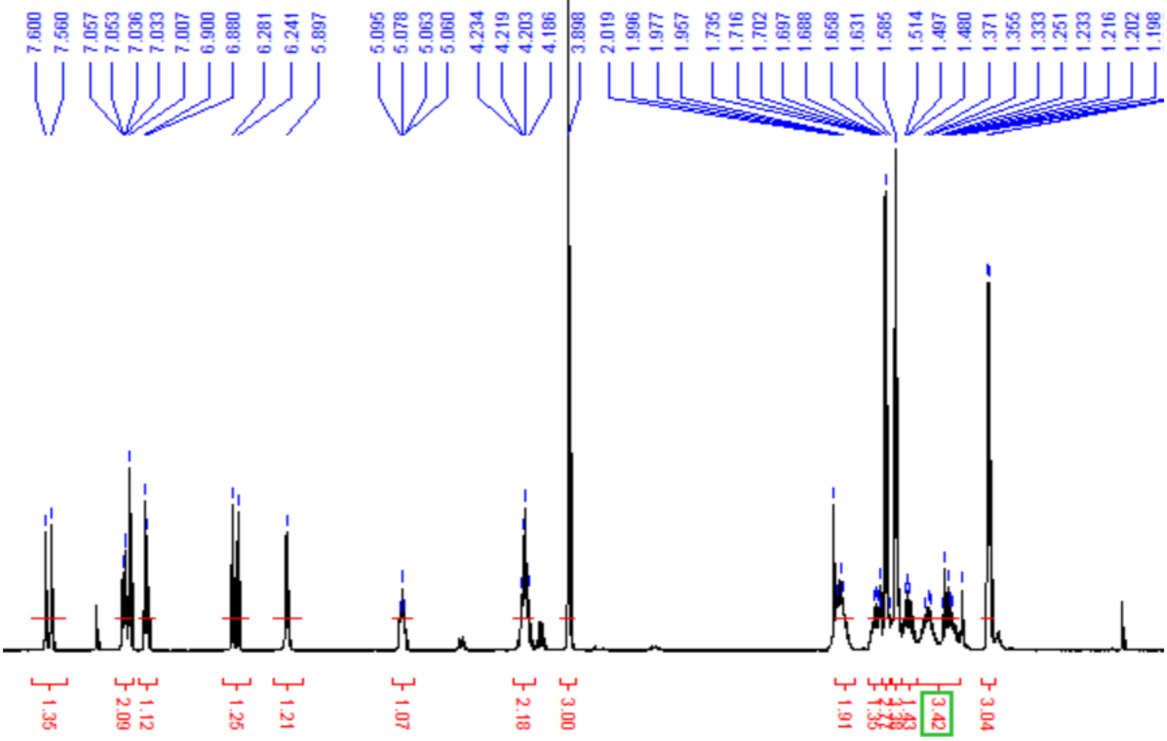

^{13}C NMR (100 MHz, $CDCl_3$)

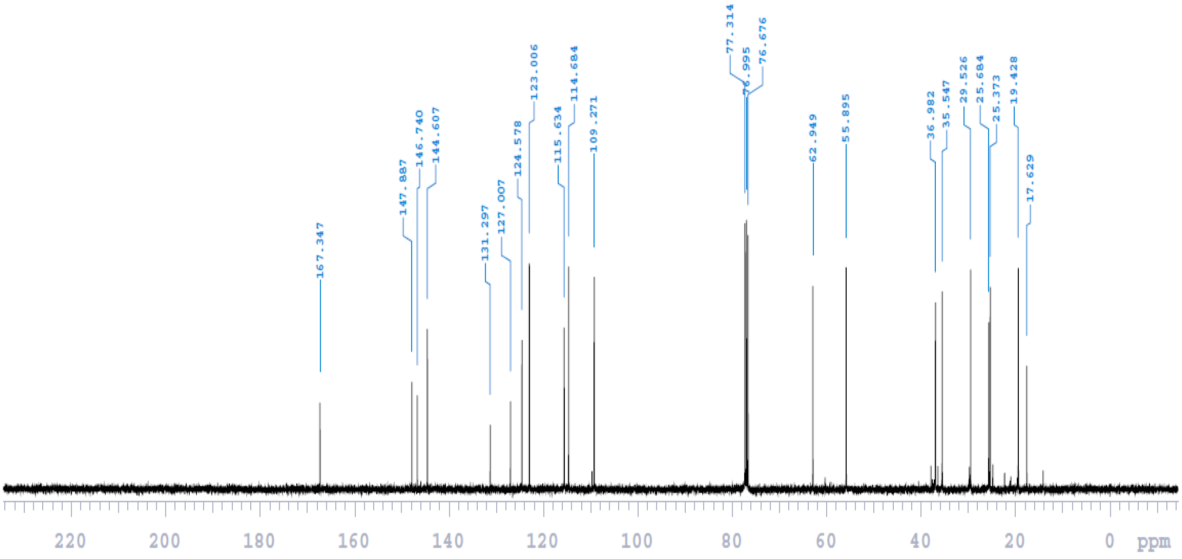

F21. Méthyl (E)-3-(3,4-diméthoxyphényl)acrylate

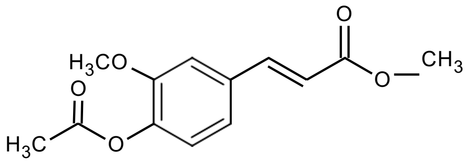

^{1}H NMR (400 MHz, $CDCl_3$)

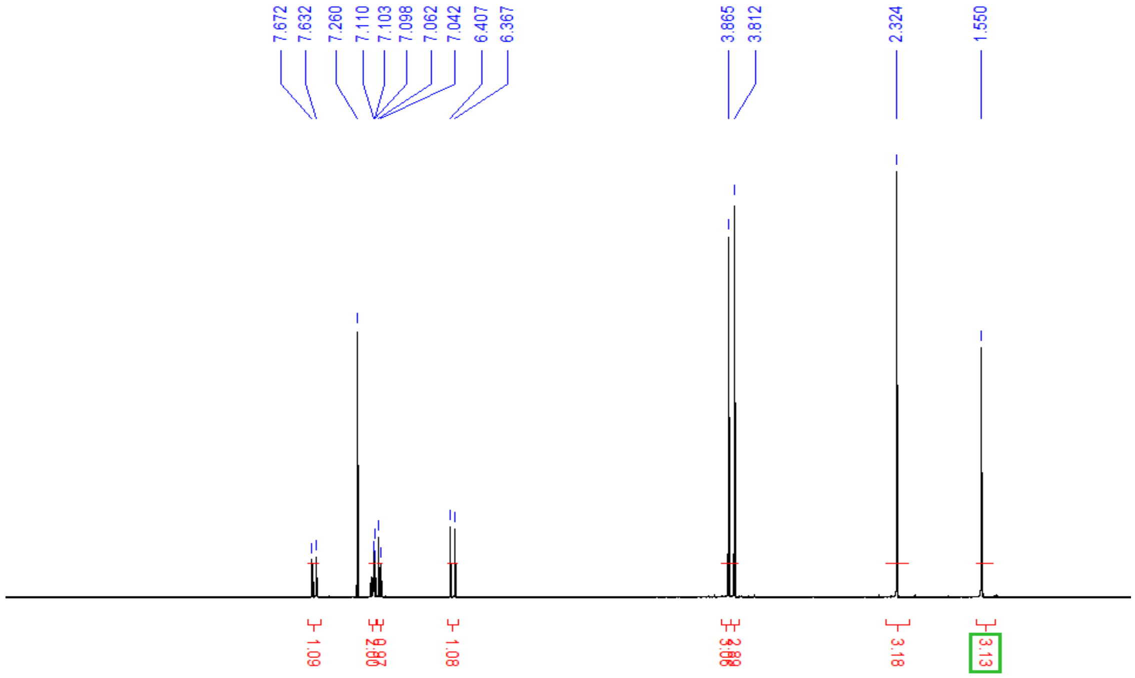

^{13}C NMR (100 MHz, $CDCl_3$)

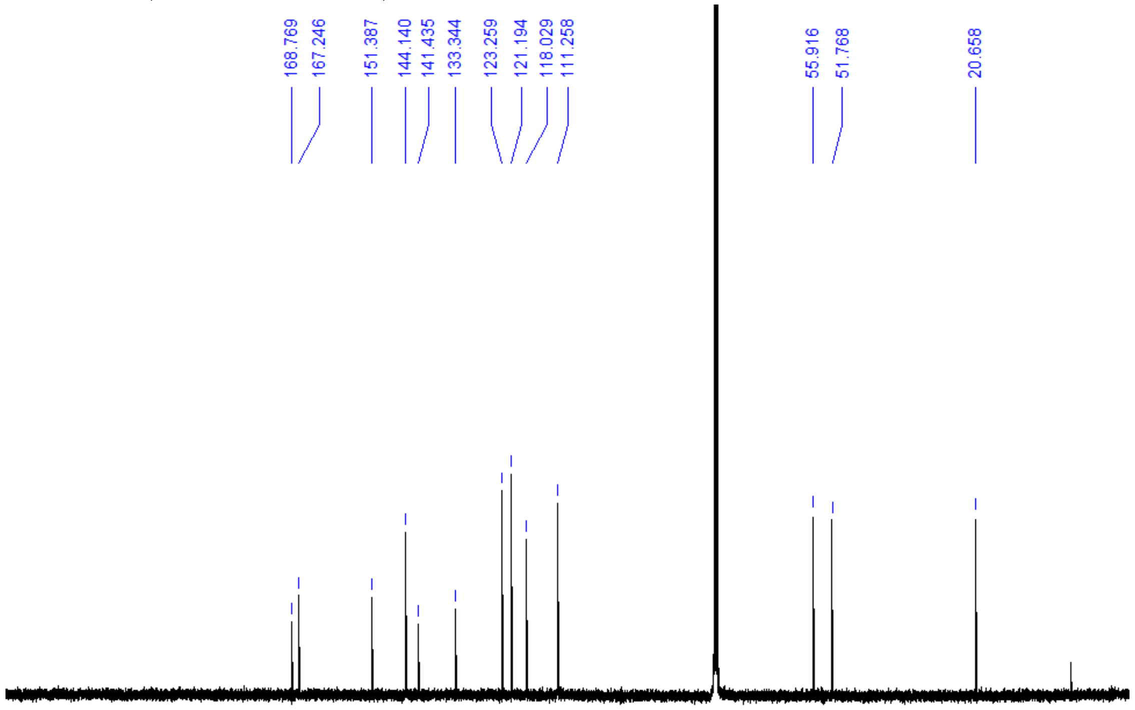

F19. (1R,2S,5R)-2-isopropyl-5-méthylcyclohéxyl (E)-3-(4-hydroxy-3-méthoxyphényl)acrylate

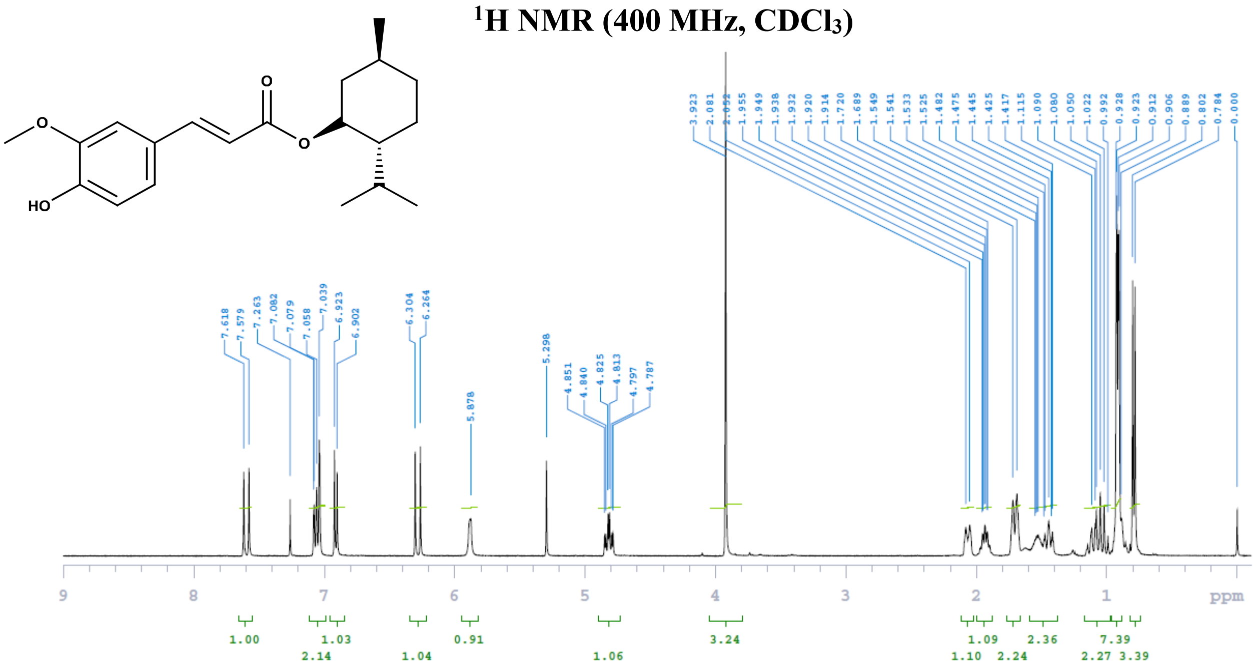

ATF19. Pentyl (2E)-3-(4-hydroxy-3,5-dimethoxyphenyl)acrylate

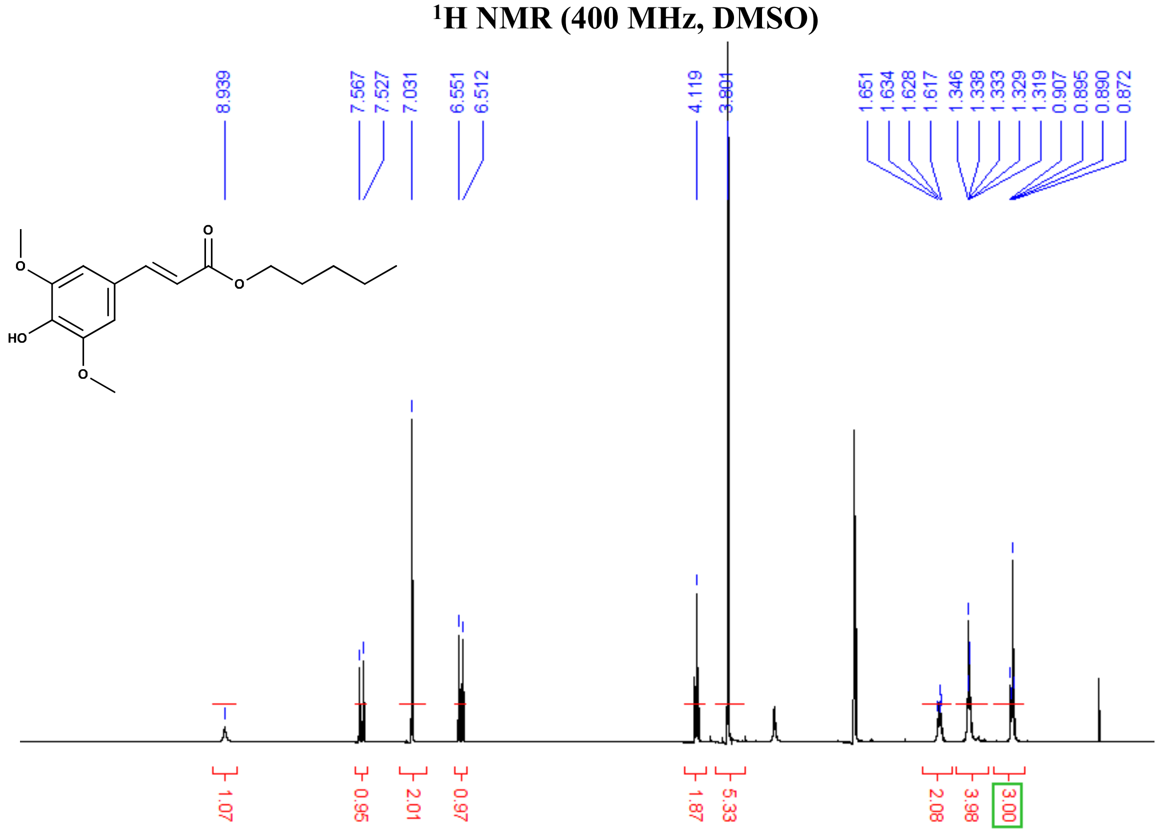

IV. Quantification of ROS production in *C. albicans*

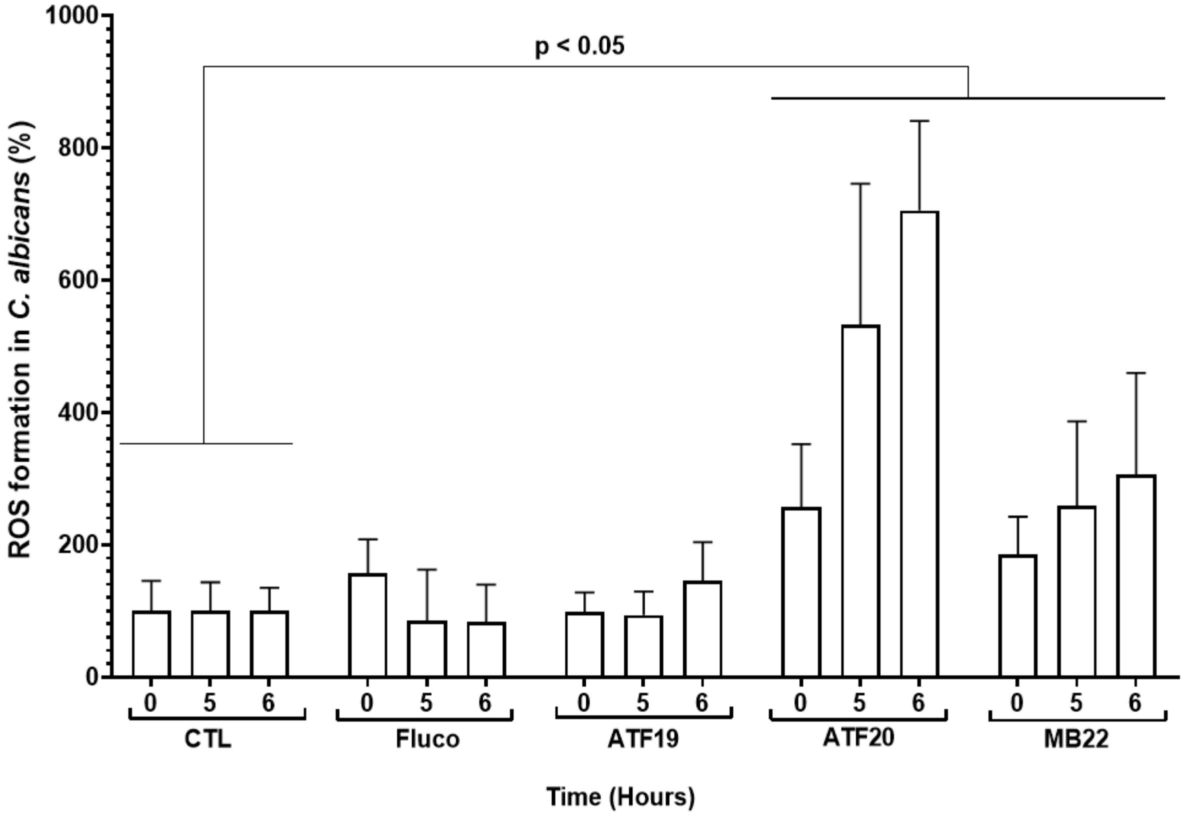

Fig. 1. Reactive oxygen species (ROS) production by *C. albicans* in the presence of ATF19, ATF20, and MB22. CTL corresponds to untreated *C. albicans* cells. Fluco refers to *C. albicans* exposed to fluconazole at 1×MIC (0.5 µg/mL). ATF19, ATF20, and ATF22 correspond to *C. albicans* exposed to each compound at 1×MIC. ROS production was measured at T0, 5 h, and 6 h.

Results

Treatment with ATF19 did not result in a significant increase in ROS production after 6 h of incubation. In contrast, exposure to ATF20 and ATF22 led to a marked elevation in ROS levels under the same experimental conditions.

Quantification of ROS production in *C. albicans*

C. albicans cells were seeded at a density of 1 × 10^5 cells/mL in a 96-well plate and incubated at 37°C for 6 h in the presence of ATF19, ATF20, ATF22, or fluconazole, each at 1×MIC. ROS production was quantified using a luminol-based chemiluminescence assay. Briefly, 50 µM luminol (97%, Sigma-Aldrich) and 5 U horseradish peroxidase (HRP; Type VI, Sigma-Aldrich) were added to each well. Luminescence was measured using a microplate reader (FLUOstar® Omega, BMG Labtech).

V. Refrences

[i] Li, N.G., Shi, Z.H., Tang, Y.P., Li, B.Q., Duan, J.A., Highly efficient esterification of ferulic acid under microwave irradiation, Molecules, **2009**, 14 (6), 2118-2126, https://doi.org/10.3390/molecules14062118.

[ii] Calheiros, R., Machado, N.F.L, Fiuza, S., Gaspar, A., Garrido, J., Milhazes, N., Borges, F., Marques, M.P.M., Antioxidant phenolic esters with potential anticancer activity : A Raman spectroscopy study, Journal of Raman Spectroscopy, **2008**, 39 (1), 95-107, https ://doi.org/10.1002/jrs.1822.

[iii] Bolling, B.W., Parkin, K.L., Phenolic Derivatives from Soy Flour Ethanol Extract Are Potent In Vitro Quinone Reductase (QR) Inducing Agents, Journal of Agricultural and Food Chemistry, **2008**, 56 (22), 10473-10480, https ://doi.org/10.1021/jf801541t.

[iv] Vafiadi, C., Topakas, E., Wong, K.K.Y., Suckling, I.D., Christakopoulos, P., Mapping the hydrolytic and synthetic selectivity of a type C feruloyl esterase (StFaeC) from Sporotrichum thermophile using alkyl ferulates, Tetrahedron : Asymmetry, **2005**, 16 (2), 373-379, https ://doi.org/10.1016/j.tetasy.2004.11.037.

[v] Roleira, F.M.F., Siquet, C., Orru, E., Garrido, E.M., Garrido, J., Milhazes, N., Podda, G., Paiva-Martins, F., Reis, S., Carvalho, R.A., Tavares da Silva, E.J., Borges, F., Lipophilic phenolic antioxidants : Correlation between antioxidant profile, partition coefficients and redox properties, Bioorganic & Medicinal Chemistry, **2010**, 18 (16), 5816-5825, https ://doi.org/10.1016/j.bmc.2010.06.090.

[vi] Yoshida, Y., Kimura, Y., Kadota, M., Tsuno, T., Adachi, S., Continuous synthesis of alkyl ferulate by immobilized Candida antartica lipase at high temperature, Biotechnology Letters, **2006**, 28 (18), 1471-1474, https://doi.org/10.1007/s10529-006-9113-8.

[vii] Katsoura, M.H., Polydera, A.C., Tsironis, L.D., Petraki, M.P., Rajacic, S.K., Tselepis, A.D., Stamatis, H., Efficient enzymatic preparation of hydroxycinnamates in ionic liquids enhances their antioxidant effect on lipoproteins oxidative modification, New Biotechnology, **2009**, 26 (1-2), 83-91, https ://doi.org/10.1016/j.nbt.2009.02.004.

[viii] Yang, C.M., Hong, J.Y., Lee, K.W., Lee, B.G., Chang, D., Preparation of kojic acid derivatives as tyrosinase inhibitors, **1995**, Patent FR2715657.

[ix] Hao, D., Yang, X.X., Zhang, J.H., Chen, G.R., Bao, Y.R., Meng, X.S., Synthesis and measuring total antioxidant ability in vitro of menthyl ferulate, Zhongguo Shiyan Fangjixue Zazhi, **2013**, 19 (15), 95-98.

[x] Kumar, V., Sharma, A. and Sinha, A. (2006), Solid -Supported Green Synthesis of Substituted Hydrocinnamic Esters by Focused Microwave Irradiation. HCA, 89: 483-495. https://doi.org/10.1002/hlca.200690049
